# Mapping Topoisomerase IV Binding and Activity Sites on the *E*. *coli* Genome

**DOI:** 10.1371/journal.pgen.1006025

**Published:** 2016-05-12

**Authors:** Hafez El Sayyed, Ludovic Le Chat, Elise Lebailly, Elise Vickridge, Carine Pages, Francois Cornet, Marco Cosentino Lagomarsino, Olivier Espéli

**Affiliations:** 1 Center for Interdisciplinary Research in Biology (CIRB), Collège de France, UMR-CNRS 7241, Paris, France; 2 Université Paris–Saclay, Gif-sur-Yvette, France; 3 Laboratoire de Microbiologie et de Génétique Moléculaires (LMGM), CNRS-Université Toulouse III, Toulouse, France; 4 UMR 7238, Computational and quantitative biology, Institut de biologie Paris Seine, Paris, France; University of Geneva Medical School, SWITZERLAND

## Abstract

Catenation links between sister chromatids are formed progressively during DNA replication and are involved in the establishment of sister chromatid cohesion. Topo IV is a bacterial type II topoisomerase involved in the removal of catenation links both behind replication forks and after replication during the final separation of sister chromosomes. We have investigated the global DNA-binding and catalytic activity of Topo IV in *E*. *coli* using genomic and molecular biology approaches. ChIP-seq revealed that Topo IV interaction with the *E*. *coli* chromosome is controlled by DNA replication. During replication, Topo IV has access to most of the genome but only selects a few hundred specific sites for its activity. Local chromatin and gene expression context influence site selection. Moreover strong DNA-binding and catalytic activities are found at the chromosome dimer resolution site, *dif*, located opposite the origin of replication. We reveal a physical and functional interaction between Topo IV and the XerCD recombinases acting at the *dif* site. This interaction is modulated by MatP, a protein involved in the organization of the Ter macrodomain. These results show that Topo IV, XerCD/*dif* and MatP are part of a network dedicated to the final step of chromosome management during the cell cycle.

## Author Summary

DNA topoisomerases are ubiquitous enzymes that solve the topological problems associated with replication, transcription and recombination. Type II Topoisomerases play a major role in the management of newly replicated DNA. They contribute to the condensation and segregation of chromosomes to the future daughter cells and are essential for the optimal transmission of genetic information. In most bacteria, including the model organism *Escherichia coli*, these tasks are performed by two enzymes, DNA gyrase and DNA Topoisomerase IV (Topo IV). The distribution of the roles between these enzymes during the cell cycle is not yet completely understood. In the present study we use genomic and molecular biology methods to decipher the regulation of Topo IV during the cell cycle. Here we present data that strongly suggest the interaction of Topo IV with the chromosome is controlled by DNA replication and chromatin factors responsible for its loading to specific regions of the chromosome. In addition, our observations reveal, that by sharing several key factors, the DNA management processes ensuring accuracy of the late steps of chromosome segregation are all interconnected.

## Introduction

DNA replication of a circular bacterial chromosome involves strong DNA topology constraints that are modulated by the activity of DNA topoisomerases [1]. Our current understanding of these topological modifications comes from extensive studies on replicating plasmids [2, 3] These studies suggest that positive supercoils are formed ahead of the replication fork, while precatenanes are formed on newly replicated sister strands. At the end of a replication round, unresolved precatenanes accumulate in the region of replication termination and are converted to catenanes between the replicated sister chromosomes. Neither precatenanes or catenanes have been directly observed on chromosomes but their presence is generally accepted and failure to resolve them leads to chromosome segregation defects and cell death [4].

Topo IV is a type II topoisomerase formed by two dimers of the ParC and ParE subunits and is the main decatenase in *Esherichia*. *coli* [5]. *in vitro*, its activity is 100 fold stronger on catenated circles than that of DNA gyrase [6]. Topo IV activity is dependent on the topology of the DNA substrate; Topo IV activity is strongest on positively supercoiled DNA and has a marked preference for L-braids, which it relaxes completely and processively. Topo IV can also unlink R-braids but only when they supercoil to form L-plectonemes [7–9]. *In vivo*, DNA gyrase appears to have multiple targets on the *E*. *coli* chromosome [10–12], whereas Topo IV cleavage sites seem to occur less frequently [11]. Interestingly, Topoisomerase IV activity is not essential for replication itself [13] but is critical for chromosome segregation [14]. The pattern of sister chromatid separation has been shown to vary upon Topo IV alteration, leading to the view that precatenanes mediate sister chromatid cohesion by accumulating for several hundred kilobases behind the replication forks keeping the newly replicated DNA together [13, 15]. The regulation of Topo IV and perhaps the accessibility of the protein to chromosome dimers was proposed to be an important factor controlling chromosome segregation [15, 16]. Topo IV activity can be modulated by a number of proteins including MukB and SeqA. MukB, is an SMC-related protein in *E*. *coli* and is reported to bind to the C-terminus of Topo IV [17] to enhance Topo IV unlinking activities [18, 19]. MukB also appears to be important in favoring the formation of Topo IV foci (clusters) near the origin of replication [20]. SeqA, a protein involved in the control of replication initiation, and Topo IV also interact [21]. These interactions may play a role in sister chromatid segregation at the late segregating SNAP regions near the origin of replication of the chromosome [16].

Beside its role in the resolution of precatenanes, Topo IV is mostly required in the post-replicative (G2) phase of the cell cycle for the resolution of catenation links. Indeed, *Espeli et al*. showed that Topo IV activity is mostly observed during the G2 phase, suggesting that a number of catenation links persist after replication [22]. Recent cell biology experiments revealed that in G2, the terminal region (*ter*) opposite *oriC* segregates following a specific pattern [23–25]. Sister *ter* regions remain associated from the moment of their replication to the onset of cell division. This sister-chromosome association is mediated by the Ter macrodomain organizing protein, MatP [26]. At the onset of cell division, the FtsK DNA-translocase processes this region, releasing the MatP-mediated association. This process ends at the *dif* site, when the dimeric forms of the sister chromosomes are resolved by the XerC and XerD recombinases. A functional interaction between the MatP/FtsK/XerCD-*dif* system and Topo IV has long been suspected. FtsK interacts with Topo IV, enhancing its decatenation activity *in vitro* [27, 28] and the *dif* region has been reported as a preferential site of Topo IV cleavage [29]. This functional interaction has been poorly documented to date and is therefore remains elusive.

In this study we have used genomic and molecular biology methods to characterize Topo IV regulation during the *Escherichia coli* cell cycle on a genome-wide scale. The present work revealed that Topo IV requires DNA replication to load on the chromosome. In addition, we have identified two binding patterns: i) regions where Topo IV binds DNA but is not engaged in a cleavage reaction; ii) numerous sites where Topo IV cleavage is frequent. We show that Topo IV-mediated removal of precatenanes is influenced by both local chromatin structure and gene expression. We also demonstrate that at the *dif* site, Topo IV cleavage and binding are enhanced by the presence of the XerCD recombinase and the MatP chromosome-structuring factor. The enhancement of Topo IV activity at *dif* promotes decatenation of fully replicated chromosomes and through interaction with other DNA management processes, this decatenation ensures accurate separation of the sister chromosomes.

## Results

### Topoisomerase IV binding on the *E*. *coli* chromosome

**Fig 1:**
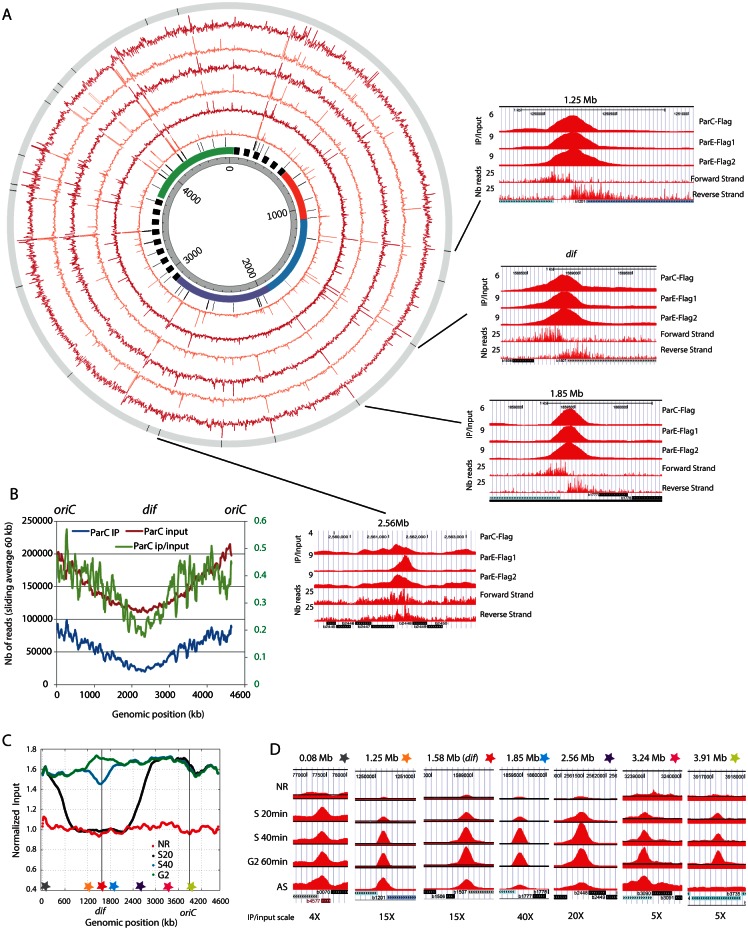
Topo IV binding pattern of replicating chromosome. A) Circos plot of the ChIP-seq experiments for ParC-flag and ParE-flag. The IP / input ratio over the entire *E*. *coli* genome is presented for three independent experiments, one IP on the *parC-flag* strain and two IPs on the *parE-flag* strain. From the center to the outside, circles represent: genomic coordinates, macrodomain map, position of tRNA genes and ribosomal operons, ParE-Flag 1 ChIP-seq (untreated data, orange), ParE-Flag 1 ChIP-seq (filtered data, red), ParE-Flag 2 ChIP-seq (untreated data, orange), ParE-Flag 2 ChIP-seq (filtered data, red), ParC-Flag ChIP-seq (untreated data, orange), ParC-Flag ChIP-seq (filtered data, red), position of the 19 validated Topo IV binding sites. The right panels represent magnifications for four specific Topo IV binding sites, position 1.25 Mb, position 1.58 Mb (*dif*), position 1.85 Mb and position 2.56Mb. The three first rows correspond to filtered IP/Input ratio for ParC-Flag, ParE-Flag1 and ParE-Flag2 IPs, the fourth and fifth rows correspond respectively to the forward and reverse raw read numbers of the *parC-flag* experiment. The position and orientation of genes are illustrated at the bottom of each panel. B) Sliding averages of the IP (blue, left Y axis), Input (red, left Y axis) and IP/input (green, right Y axis) data for the *parC-flag* experiment over 60 kb regions along the genome. To facilitate the reading, *oriC* is positioned at 0 and 4.639 Mb. C) Analysis of Topo IV binding during the bacterial cell cycle. Marker frequency analysis was used to demonstrate the synchrony of the population at each time point. Stars represent the position of the selected Topo IV sites. D) IP/input ratio for 7 regions presenting specific Topo IV enrichment during S and G2 phases. For each genomic position the maximum scale is set to the maximum IP/Input ratio observed.

To identify Topo IV binding, we performed ChIP-seq experiments in ParE and ParC Flag tagged strains. The C-terminus fusions of ParE and ParC replaced the wild-type (WT) alleles without any observable phenotypes (S1 Fig). We performed three independent experiments, two ParE-flag IPs and one ParC-flag IP, with reproducible patterns identified in all three experiments. A Pearson correlation of 0.8, 0.9 and 0.7 was observed for ParC-ParE1, ParE1-ParE2 and ParC-ParE2 respectively. A map of enriched regions observed in each experiment is represented on Fig 1A (red circles). Four of the highly-enriched sites are illustrated at a higher magnification in Fig 1A—right panels. Interestingly one of these sites corresponds to the *dif* site (position 1.58Mb), which has previously been identified as a strong Topoisomerase IV cleavage site in the presence of norfloxacin [29]. We also observed strong enrichment over rRNA operons, tRNA and IS sequences. To address the significance of the enrichment at rRNA, tRNA and IS, we monitored these sites in ChIP-seq experiments performed in the same conditions with a MatP-flag strain and mock IP performed with strain that did not contain any flag tagged protein. Both MatP and Mock IP presented significant signals on rRNA, tRNA and IS loci (S2 Fig). This observation suggested that Topo IV enrichment at rRNA, tRNAs and IS was an artifact of the ChIP-Seq technique. By contrast no enrichment was observed at the *dif* site in the MatP and mock-IP experiments (S2 Fig), we therefore considered *dif* to be a genuine Topo IV binding site and compared every enriched region (>2 fold) with the *dif* IP. We filtered the raw data for regions presenting the highest Pearson correlation with the *dif* signal (>0.7). This procedure discarded many highly enriched regions (Fig 1A orange circles). We identified 19 sites throughout the chromosome where Topo IV IP/input signal suggested a specific binding for at least two of the experiments (Fig 1A, outer circle histogram, S1 Table). Most Topo IV binding sites span a 200 bp region. These sites frequently overlapped intergenic regions, with their mid-points located inside the intergenic region, and did not correlate with any identifiable consensus sequence. In addition to *dif*, which exhibited a 10-fold enrichment, three other sites were strongly enriched. These sites corresponded to positions 1.25Mb (9.4x), 1.85Mb (31x) and 2.56Mb (19x) on the chromosome (Fig 1A, right panels). Beside these specific sites, Topo IV IP showed non-specific enrichment in the *oriC* proximal half of the chromosome. This bias was not a consequence of locus copy number, as the enrichment remained after copy number normalization (Fig 1B). We used MatP-Flag IP [30] and a control IP in a strain that does not contain a Flag tagged gene to differentiate non-specific Topo IV binding from experimental noise (S3A Fig). In addition, Topo IV enrichment was also observed in GC rich regions of the chromosomes (S3B Fig). Importantly, the *ori/ter* bias was not a result of the GC% bias along the chromosome since it was still explicit after GC% normalization (S3C Fig). More precisely, the Topo IV binding pattern closely followed gene dosage for a ~3Mb region centered on *oriC* (S3D and S3E Fig and S1 Text). In the complementary ter-proximal region, gene dosage (input reads) was higher than the ChIP-seq profile, suggesting that the nonspecific Topo IV binding was lower or lasts for a shorter time in the cell cycle (since these data are population-averaged). The Terminus region that is depleted in Topo IV binding (1.6Mb) surpassed, by far, the size of the Ter macrodomain (800kb).

### Topo IV binding is influenced by replication

The influence of Topo IV on sister chromatid interactions [15] prompted the question of how Topo IV would follow replication forks and bind to the newly replicated sister chromatids throughout the cell cycle. We performed ChIP-seq experiments in *E*. *coli dnaC2* strains under conditions suitable for cell cycle synchronization of the entire population. Synchronization was achieved through a double temperature shift, as described previously [15]. Using these conditions, in each cell, S phase is initiated on one chromosome, lasts for 40–45 min and is followed by a G2 phase (20 min) (S4 Fig). We analyzed ParE binding before the initiation of replication, in S phase 20 min (S20) and 40 min (S40) after the initiation of replication and in G2 phase. The synchronization of replication in the population was monitored by marker frequency analysis of the Input DNA (Fig 1C). The profile observed for bacteria that did not replicate at non-permissive temperature was strictly flat, but the S20 replication profile presented two sharp changes of the marker frequency slope around positions 500kb and 2700kb. This suggested that each replication fork had crossed approximately 1000 to 1300 kb in 20 min. The S40 replication profile demonstrated that most cells had finished replication, with the unreplicated region being limited to 300 kb around *dif* in no more than 20% of the bacteria. In G2 phase the marker frequency was flat. We used flow cytometry to demonstrate that at G2, the amount of DNA in each bacterium was double compared to that of the G1 bacteria, indicating that cytokinesis has not yet occurred (S4 Fig). We analyzed Topo IV binding at specific binding sites (Fig 1D). Binding at these sites was strongly impaired in the absence of replication. Binding at every site started in the S20 sample and was maximal in the S40 or G2 samples, without showing any marked decrease, even in the *oriC*-proximal region. These observations suggest that Topo IV binds to specific sites during S phase. However, since enrichment was observed for non-replicated loci and was maintained for a long time after replication, it was not compatible with a model of Topo IV migration with the replication forks. Synchronization experiments with a higher temporal resolution are required to clarify this observation.

### Only certain Topo IV binding sites correspond to Topo IV cleavage sites

**Fig 2:**
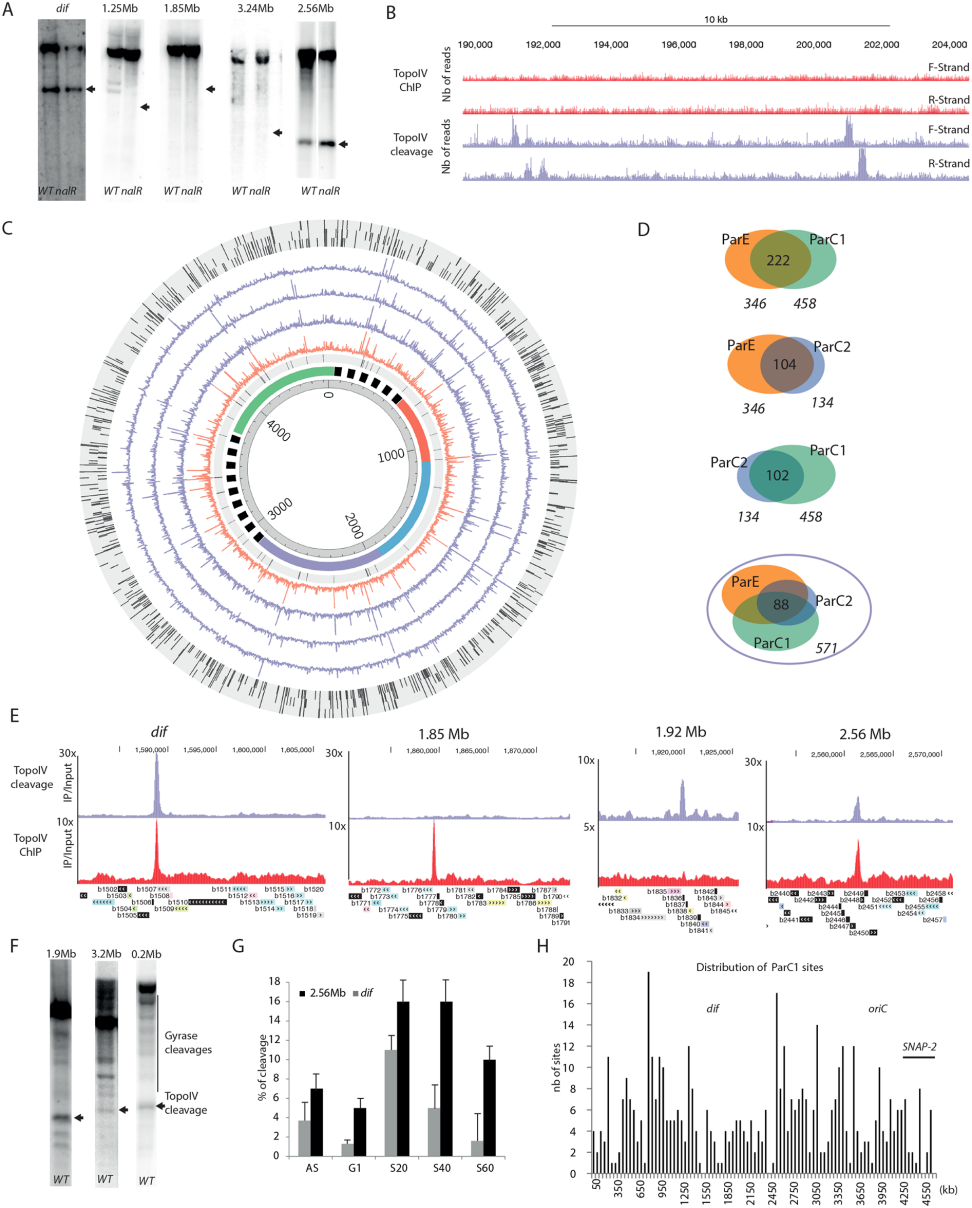
Topo IV cleavage at the Topo IV binding sites. A) Norfloxacin mediated DNA cleavage revealed by Southern blot with a radiolabeled probe near the *dif* site, the 1.25 Mb, 1.85 Mb, 2.56 Mb and the 3.24 Mb site. The size of the expected fragment generated by Topo IV cleavage is marked by an arrow. Topo IV cleavage can be differentiated from gyrase cleavages because of their presence in a *nalR* strain. B) Genome browser image of a 15kb region representative of Topo IV cleavage frequency (purple). These cleavage sites are not correlated with Topo IV enrichment in the ChIP-seq experiments described in Fig 1 (red). C) Circos plot of the NorflIP experiments. From the center to the outside, circles represent: genomic coordinates, macrodomain map, position of tRNA genes and ribosomal operons, ParC-Flag 1 NorflIP (untreated data, orange), ParC-Flag 1 NorflIP (filtered data, purple), ParE-Flag NorflIP(filtered data, purple), ParC-Flag 2 NorflIP (filtered data, purple), validated TopoIV sites present in the ParC-Flag 1, ParE-Flag and ParC-Flag 2 experiments. For visualization purpose, the maximum scale of NorflIP data has been fixed to an IP/input ratio of 10. D) Peak calling procedure, dedicated to DNA cleavage mediated by TopoIV in the presence of norfloxacin (S6 Fig), revealed 571 sites in total, in three experiments. Venn diagrams of common Topo IV cleavage sites in two experiments. About 200 common sites are observed in each pair of experiments. E) Genome browser zooms on the *dif*, 1.85, 1.92 and 2.56 Mb regions for Topo IV cleavage (purple) and Topo IV binding revealed by ChIP-seq (red). F) DNA cleavage mediated by TopoIV in the presence of norfloxacin revealed by Southern blot with a radiolabeled probe at 0.02, 1.92 Mb and the 3.2 Mb sites. G) Cleavage experiments performed on synchronized cultures, revealed a replication dependency (AS asynchronous, NR not replicating, S20 20 min after the initiation of replication (IR), S40 40 min after IR, S60 60 min after IR. H) Distribution of the ParC-Flag 1 NorflIP validated sites on the genome by 50 kb bins.

To measure Topo IV cleavage at the binding sites, we took advantage of the fact that norfloxacin covalently links Topoisomerase II to the gate segment of DNA and prevent its relegation [31]. We first monitored Topo IV activity on the Topo IV enriched regions (1.2, 1.8, 2.5, 3.2 Mb and *dif*) by incubating bacteria with norfloxacin for 10 minutes before genomic extraction and performing Southern blot analysis to detect the cleaved DNA products [10, 29]. This revealed cleavage fragments induced by both DNA Gyrase and Topo IV poisoning in the WT strain, but only Topo IV cleavage in a *nalR* strain where DNA Gyrase is resistant to norfloxacin. Among the 5 tested sites, only two displayed clear Topo IV cleavage at the expected position (Fig 2A). As expected, the *dif* site exhibited strong cleavage. Moreover cleavage was also observed at position 2.56 Mb. However the 1.2, 1.8 and 3.2 Mb sites did not show any Topo IV mediated cleavage in the presence of norfloxacin.

### Topo IV presents hundreds of cleavage sites on the chromosome

The above result prompted us to investigate Topo IV cleavage at the genome-wide scale. We performed IPs in the presence of norfloxacin as a crosslinking agent instead of formaldehyde. Following this step, all downstream steps of the protocol were identical to that of the ChIP-Seq assay. We referred to this method as NorflIP. The NorflIP profile differed from the ChIP-seq profile (Fig 2B). Regions immunoprecipitated with Topo IV-norfloxacin cross-links were frequently observed (Fig 2C orange circle). Similarly to the ChIP-seq experiments, the NorflIP profile revealed strong enrichment over the rRNA operons and IS sequences but not at the tRNA genes (S5A Fig). We used a Southern blot cleavage assay to demonstrate that these signal did not correspond to Topo IV cleavages (S5B Fig). The NorflIP peaks correspond to a ~170 bp forward and reverse enrichment signal separated by a 130 bp segment, which is not enriched. This pattern is the consequence of the covalent binding of Topo IV to the 5’ bases at the cleavage site. After Proteinase K treatment the cleaving tyrosine residue bound to the 5’ extremity resulted in poor ligation efficiency and infrequent sequencing of the cleaved extremities. (S6A and S6B Fig) This observation confirmed that we were observing genuine Topoisomerase cleavage sites. We used this pattern to define an automatic peak calling procedure (S6C Fig) that identified between 134 and 458 peaks in the three NorflIP experiments, two experiments performed with ParC-Flag and one with ParE-Flag (Fig 2C purple circles and Fig 2D). We observed a total of 571 possible sites in the three experiments with about half of the sites common to at least two experiments and approximately 88 sites common to all three experiments (S1 Table). We analyzed sequencing reads for the three experiments around the *dif*, 0.2 Mb and 1.92Mb positions. It revealed abrupt depletions of forward and reverse reads in a 100bp center region suggesting that it corresponds to the site of cleavage. We extrapolated this result for every peak to estimate the cleavage positioning of Topo IV (~150bp downstream of the center of the forward peak, S6D Fig) We manually validated 172 sites that were common to ParC-1 and ParE-1 experiments (S1 Table) for further analysis.

### Characteristics of Topo IV cleavage sites

The Topo IV cleavage at the *dif* site was the most enriched of the chromosome (~ 30 fold), fourteen sites were enriched from 5 to 10 fold and other positions were enriched from 2 to 5 fold (Fig 2E). Most NorflIP sites did not correspond to significant peaks in the ChIP-seq experiment (Fig 2E). We also did not observe any cleavage for the majority of the strong binding sites observed by ChIP-seq. This is illustrated for the binding site at 1.85 Mb (Fig 2E). We verified several Topo IV cleavage sites by Southern blot, a significant cleaved DNA fragment was observed at the expected size for each of them (Fig 2F). Southern blotting experiments following DNA cleavage in the presence of norfloxacin on synchronized cultures revealed that, like its binding, Topo IV cleavage is coordinated with DNA replication. In good agreement with ChIP-seq experiments, increased cleavage was observed as soon as 20 minutes after initiation of replication for the *dif* and 2.56 Mb sites (Fig 2G).

### Genomic distribution of Topo IV cleavage sites

The general genomic distribution of Topo IV cleavage sites was not homogeneous; a few regions had a large number of sites clustered together, while the 1.2Mb– 2.5 Mb region contained a low density of sites (Fig 2H). We further analyzed the distribution of cleavage sites in the terminus and the *oriC* regions. In the terminus region, the average distance of consecutive cleavage sites was long (around 30 kb in the 1.5–2.5 Mb region) compared to 8 kb in the 0.8–1.5 Mb or the 2.5–3.1 Mb regions (S7A Fig). The *oriC* region displays a mixed distribution (S7B Fig), a high density of sites near *oriC* flanked by two depleted regions, including the SNAP2 region [16]. At the gene scale, the mid-point of Topo IV cleavage signal can be localized inside genes (82%) or intergenic regions (16%) but it presents a bias toward the 5’ or 3’ gene extremities (S7C Fig). Since the cleavage signal spans approximately 200bp, nearly 50% of the sites overlapped, at least partly, with intergenic regions that account for only 11% of the genome. Finally, we did not identify any robust consensus between sets of Topo IV cleavage sites. The only sequence traits that we identified are a bias for GC dinucleotides near the center of the sites (S7D Fig) and an increased spacing of GATC motifs around cleavage sites (S7E Fig).

### Targeting of Topo IV cleavage activity is influenced by local environment

**Fig 3:**
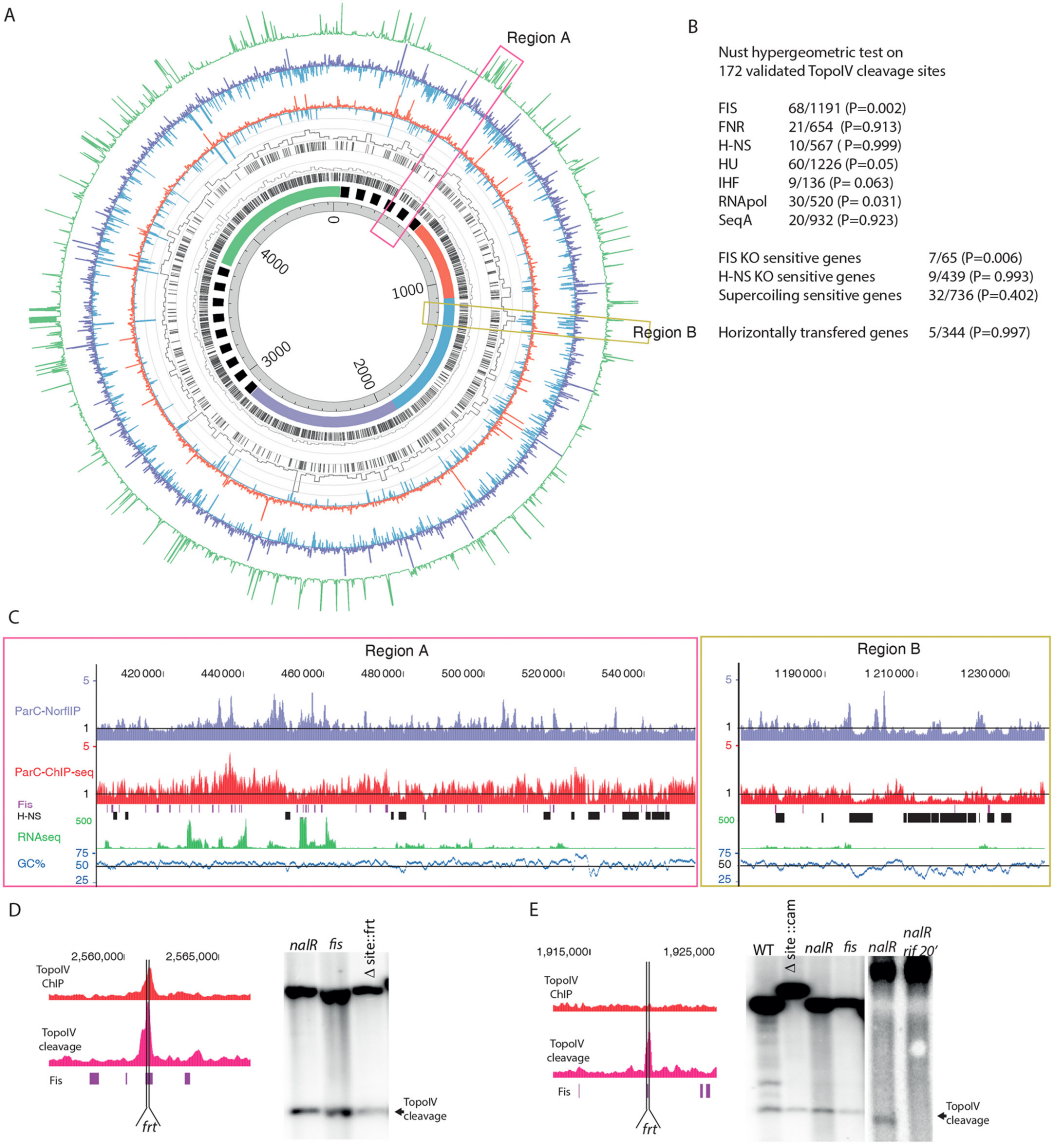
Targeting Topo IV cleavage sites along the *E*. *coli* genome. A) Circos plot of the ParC-Flag Chipseq and ParC-Flag 1 NorflIP experiments. From the center to the outside, circles represent: genomic coordinates, macrodomain map, Fis binding sites in mid exponential phase, % of bases bound by Fis per 20 kb windows of genomic DNA, H-NS binding sites in mid exponential phase, % of bases bound by H-NS per 20 kb windows of genomic DNA [33], ParC-Flag ChIP-seq (depleted regions blue, IP/input <1), ParC-Flag ChIP-seq (enriched regions red, IP/input >1), ParC-Flag 1 NorflIP (depleted regions blue, IP/input <1), ParC-Flag 1 NorflIP (enriched regions red, IP/input >1), gene expression data (RNA-seq results performed in the ChIP-seq and NorflIP conditions). For visualization purpose, the maximum scale of RNAseq data has been fixed to 500 reads which approximately corresponds to the 400 transcription units that were the most expressed (the distribution of read counts scaled from 0 to 30 000). B) Correlation between the localization of Topo IV cleavages and chromatin markers. The NUST [32] hypergeometric test was used to compare Topo IV and chromatin markers localization. The set of 172 validated Topo IV cleavage sites was used. The number of common localizations over the total number of chromatin marker localization is indicated. The P value of a Fisher’s exact test is indicated. C) Genome browser magnifications of the panel A’s pink and yellows regions. Mid log phase Fis and H-NS binding sites are respectively indicated with burgundy and black boxes [33]. D) Magnification of the 2.56 Mb Topo IV binding and cleavage site that overlaps a Fis binding site. The position of the deleted Topo IV site is marked by vertical lines (*frt*). Southern blot analysis of Topo IV cleavage at the 2.56 Mb locus, in the *nalR strain*, the *nalR* strain with a deletion of the Topo IV cleavage and binding site and the deletion of *fis*. E) Same as D for the 1.92 Mb Topo IV cleavage site. Southern blot analysis of Topo IV cleavage at the 1.92 Mb locus, in the WT, the *nalR strain*, the *nalR* strain with a deletion of the Topo IV cleavage and Fis binding site and the *nalR* strain with *fis* deletion. The cleavage was also analyzed following a 20 min treatment with rifampicin (rif).

The bias in the distribution of cleavage sites (Fig 2H) was very similar to the Topo IV binding bias revealed by ChIP-seq (Fig 1C). NorflIP and ChIP-seq data were compared on Fig 3A. Despite the lack of corresponding ChIP-seq enrichment at the position of most highly enriched NorflIP sites, a number of consistencies were observed between these two data sets. Overall the NorflIP and ChIP-seq datasets had a Pearson correlation of 0.3 and the averaged data (1 kb bin) revealed a Pearson correlation of 0.5. First a small amount of local enrichment in the ChIP-seq experiments was frequently observed in the regions containing many cleavage sites (Fig 3A and 3C). This led us to consider that trapped Topo IV engaged in the cleavage reaction could contribute to a small amount of local enrichment in the ChIP-seq experiments. Second, both Topo IV cleavages and binding sites were rare in highly expressed regions (Fig 3A), only one of the 172 manually validated Topo IV cleavage site overlapped a highly expressed region. However cleavages sites were more frequently, than expected for a random distribution, observed in their vicinity (Fig 3C and S8 Fig). Thirty percent (50/172) of the Topo IV sites are less than 2 kb away from the next highly expressed transcription unit (Fig 3).

We explored correlations between the localization of Topo IV cleavages and binding sites of various NAPs thanks to the Nust database and tools [32]. A significant correlation was only observed for Fis binding sites (Fig 3B). Sixty eight genes present both Fis binding [33] and Topo IV cleavage (P value 2x10^-03^). Thirty-three of the 172 manually validated cleavage sites overlapped at least partially with a Fis binding site, 80 of them are located less than 400 bp away from a Fis binding site. At the genome scale this correlation is difficult to observe (Fig 3A), but close examination clearly revealed overlapping Topo IV cleavages and Fis binding sites (Fig 3C). Fis binding sites are more numerous than Topo IV cleavage sites, therefore a large number of them do not present enrichment for Topo IV (Fig 3C). By contrast, Topo IV peaks are excluded from H-NS rich regions (Fig 3A, 3B and 3C). Only one of the 172 manually validated Topo IV cleavage site overlapped with an H-NS binding site. As observed for highly expressed regions TopoIV cleavage sites were frequently observed at the border of H-NS rich regions (Fig 3C). Moreover H-NS rich regions contain less Topo IV than the rest of the chromosome (Fig 3A–3D and S9A Fig). H-NS rich regions correspond to an AT rich segment of the chromosome (Fig 3C and 3D). Indeed background level of Topo IV binding and cleavage were significantly reduced in AT rich regions (S9B Fig). In rare occasions binding of H-NS has been observed in regions with a regular AT content (Fig 3C), notably Topo IV binding and cleavage were also reduced in these regions. This observation suggested that H-NS itself rather than AT content limits the accessibility of Topo IV to DNA. This observation was confirmed by the identification of Topo IV cleavage in regions with an AT content ranging from 20 to 80% (S9C and S9D Fig).

We performed Southern blot analysis of Topo IV cleavage on representative sites to test whether gene expression and chromatin factors influenced Topo IV site selection. First, we observed that the exact deletion of cleavage sites at position 1.92 Mb and 2.56 Mb did not abolish Topo IV cleavage activity (Fig 3D and 3E). Second, since these loci also contain a Fis binding site overlapping Topo IV cleavage signal, we deleted the *fis* gene. However, deletion of the *fis* gene did not modify Topo IV cleavage (Fig 3D and 3E). Finally we performed cleavage assays in the presence of rifampicin to inhibit transcription. To limit the pleiotropic effects of rifampicin addition we performed the experiment with a 20 min pulse of rifampicin. Rifampicin treatment abolished Topo IV cleavage (Fig 3E). These results suggest that gene expression rather than chromatin factors influences Topo IV targeting.

### XerC targets Topo IV to the *dif* site

**Fig 4:**
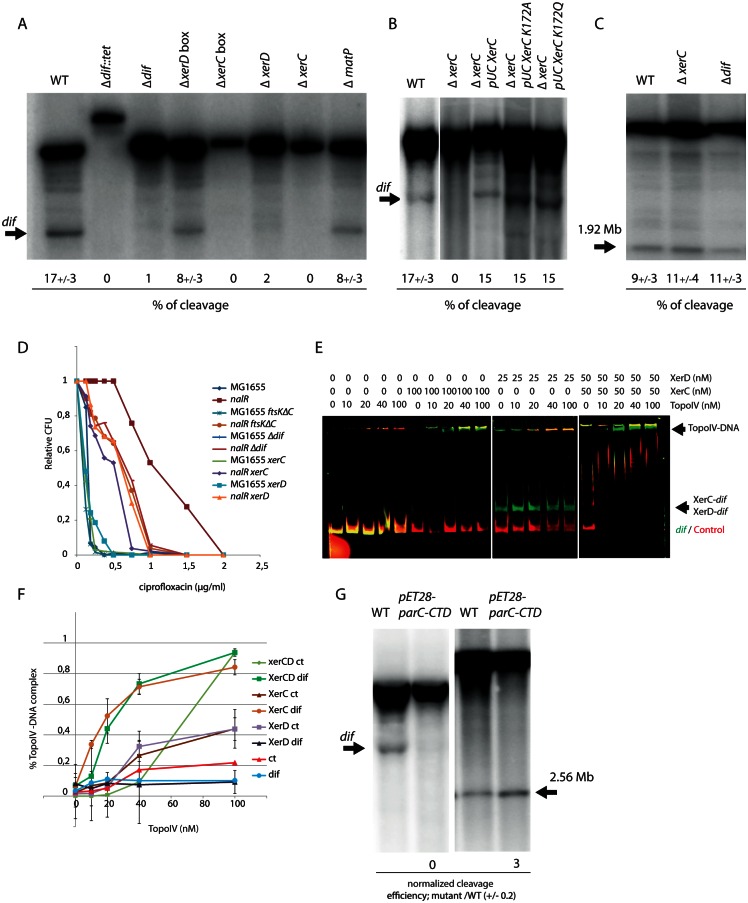
Determinants of Topo IV activity at *dif*. A) Southern blot analysis of the Topo IV cleavage at the *dif* site. Genomic DNA extracted from WT, Δ*dif*::*Tc*, Δ*dif*, Δ*xerD* box, Δ*xerC* box, Δ*xerD*, and Δ*xerC* strains was digested by *Pst*I; the size of the fragment generated by Topo IV cleavage at *dif* is marked by an arrow. The average percentage of cleavage observed in two independent experiments is presented. B) Southern blot analysis of the Topo IV cleavage at the *dif* site. Genomic DNA extracted from WT, Δ*xerC*, Δ*xerC pUCxerC*, Δ*xerC pUCxerCK172A*, Δ*xerC pUCxerCK172Q* strains was digested with *Pst*I; the size of the cleaved fragment in *dif* is marked by an arrow. C) Topo IV cleavage at the 1.9Mb site in the WT, Δ*xerC and* Δ*dif*. D) Plating of *parEts*, *parEts xerC*, *parEts xerD and parEts recN* mutants at 30 and 37°C. E) Colony Forming Unit (CFU) analysis of the WT and *nalR* strains deleted for the *dif* site, the *xerC*, *xerD* genes or the C-terminal domain of FtsK in the presence of ciprofloxacin. F) EMSA on a 250 bp CY3 probe containing *dif* (green) and a 250 bp CY5 control probe (red). The amount of Topo IV, XerC or XerD proteins present in each line is indicated above the gel. G) Quantification of Topo IV EMSA presented in C, data are an average of three experiments. H) Southern blot analysis of Topo IV cleavage at *dif* and position 1.92Mb in a strain overexpressing the C-terminal domain of ParC.

Our analysis confirms that the *dif* region is a hot spot for Topo IV activity [29]. Indeed, ChIPseq and NorflIP show that Topo IV binds to and cleaves frequently in the immediate proximity of *dif*. We measured DNA cleavage by Topo IV in the presence of norfloxacin in various mutants affecting the structure of *dif* or genes implicated in chromosome dimer resolution. Southern blot was used to measure Topo IV cleavage (Fig 4A). We observed that exact deletion of *dif* totally abolished Topo IV cleavage. Interestingly, the deletion of the XerC-binding sequence (XerC box) of *dif* was also sufficient to abolish cleavage, while the deletion of the XerD box only had a weak effect. Deletion of the *xerC* and *xerD* genes abolished Topo IV cleavage at *dif*. However, cleavage was restored when the catalytically inactive mutants XerC K172A or XerC K172Q were substituted for XerC (Fig 4B). This suggests that the role of XerCD/*dif* in the control of Topo IV activity is structural and independent of XerCD catalysis. Deletion of *dif* or *xerC* did not significantly alter cleavage at any of the other tested Topo IV cleavage sites (Fig 4C). This suggests that influence of XerC on Topo IV is specific to *dif*.

To evaluate the role of XerCD-mediated Topo IV cleavage at *dif*, we attempted to construct *parEts xerC*, *parEts xerD* and *parCts xerC* double mutants. We could not obtain *parCts xerC* mutants by P1 transduction at any tested temperature. We obtained *parEts xerC* and *parEts xerD* mutants at 30°C. The *parEts xerC* double mutant presented a growth defect phenotype at 30°C and did not grow at temperature above 35°C (Fig 4D). The *parEts xerD* mutant presented a slight growth defect at 37°C compared to *parEts* or *xerD* mutants. None of the *parEts* mutant grew above 42°C. Next, we used quinolone sensitivity as a reporter of Topo IV activity. To this aim, we introduced mutants of the FtsK/Xer system into a *gyrA*^*nalR*^ (*nalR*) strain; Topo IV is the primary target of quinolones in such strains. The absence of XerC, XerD, the C-terminal activating domain of FtsK or *dif* exacerbated the sensitivity of the *nalR* strain to ciprofloxacin (Fig 4D). We therefore concluded that the impairment of Topo IV was more detrimental to the cell when the FtsK/Xer system was inactivated. Among partners of the FtsK/Xer system the absence of XerC was significantly the most detrimental, suggesting a specific role for XerC in this process.

The above results suggest an interaction between Topo IV and the XerCD/*dif* complex. We therefore attempted to detect this interaction directly *in vitro* (Fig 4E and 4F). We performed EMSA with two fluorescently labeled linear probes, one containing *dif* and the other containing a control DNA not targeted by Topo IV in our genomic assays. Topo IV alone bound poorly to both probes (Kd > 100nM). Binding was strongly enhanced when XerC or both XerC and XerD were added to the reaction mix. In contrast, Topo IV binding to *dif* was slightly inhibited in the presence of XerD alone. These results were consistent with the observation that deletion of the XerC box but not of the XerD box inhibited Topo IV cleavage at *dif* and pointed to a specific role for XerC in Topo IV targeting. The control fragment showed that these effects are specific to *dif*. Topo IV-XerC/*dif* complexes were stable and resisted a challenge by increasing amount of XerD (S10A Fig). The positive influence of XerCD on TopoIV binding was also observed on a negatively supercoiled plasmid containing *dif*. In the presence of XerCD (50nM), a delay in the plasmid migration was observed with 40nM of TopoIV. By contrast, 200 nM was required in the absence of XerCD (S10B Fig). The Southern blot cleavage assay showed that overexpression of the ParC C-terminal domain (pET28parC-CTD) strongly reduced cleavage at *dif* but enhanced cleavage at the Topo IV site located at 2.56Mb. This suggested that, as observed for MukB [17], Topo IV might interact with XerC through its C-terminal domain (Fig 4G).

### Topo IV activity at *dif* depends on dynamics of the *ter* region and chromosome circularity

**Fig 5:**
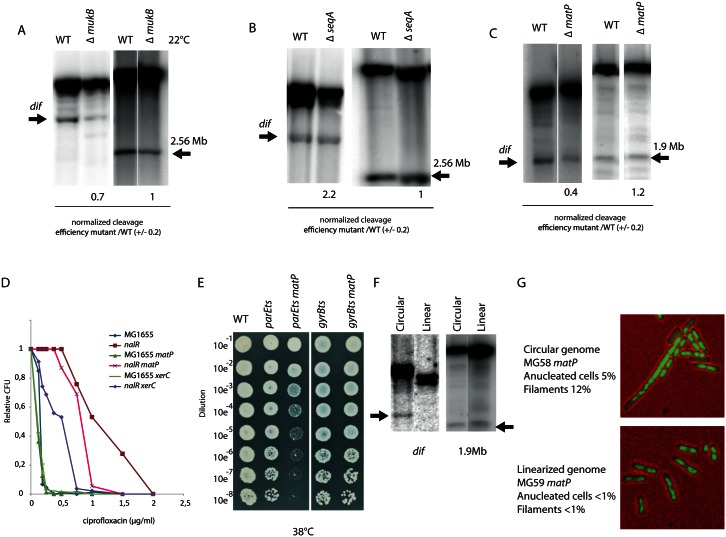
Role of the *dif* site for the management of circular chromosomes. A) Southern blot analysis of Topo IV cleavage at the *dif* and 2.56 Mb sites in the *mukB* mutant grown in minimal medium at 22°C. B) Southern blot analysis of Topo IV cleavage at the *dif* and 2.56 Mb sites in the *seqA* mutant grown in minimal medium at 37°C. C) Southern blot analysis of Topo IV cleavage at the *dif* and 1.9 Mb sites in the *matP* mutant grown in LB at 37°C. D) Colony Forming Unit (CFU) analysis of the WT and *nalR* strains deleted for the *dif* site, the *xerC* and *matP* genes in the presence of ciprofloxacin. E) Colony Forming Unit (CFU) analysis of the WT, *parEts* and *gyrBts* strains deleted for the *matP* at a semi permissive temperature (38°C). F) Southern blot analysis of the Topo IV cleavage at the *dif* and 1.9Mb sites in cells with a circular or linearized chromosome. G) Phenotypes observed during exponential growth in LB in the *matP* mutant strains with circular or linear chromosome (DNA is labeled with DAPI, green). Scale bar is 5μm.

We assayed the effects of the reported Topo IV modulators and proteins involved in chromosome segregation the activity of Topo IV at *dif*. MukB has previously been shown to influence the activity of Topo IV [17, 18]. We measured Topo IV cleavage in a *mukB* mutant at *dif* and at position 2.56 Mb, cleavage was reduced at *dif* but no significant effect was observed at position 2.56Mb (Fig 5A). We did not detect any effect of a *seqA* deletion on Topo IV cleavage at either position (Fig 5B). We next assayed the effect of MatP, which is required for compaction and intracellular positioning of the *ter* region as well as for the its progressive segregation pattern ending at *dif* [25, 26]. The Topo IV cleavage at *dif* was significantly impaired in the *matP* mutant (Fig 5C). The Topo IV cleavage site at position 1.9Mb is included in the Ter macrodomain, but cleavage at this site was almost unchanged in the absence of MatP (Fig 5C). Introduction of a *matP* deletion into the *nalR* strain yielded an increase in ciprofloxacin sensitivity (Fig 5D). We also constructed a *parEts matP* double mutant. Growth of this strain was significantly altered compared to the *parEts* parental strain at an intermediate temperature (Fig 5E). Such a synergistic effect was not found when combining the *matP* deletion with a *gyrBts* mutation. Taken together, these results led us to consider that MatP itself or the folding of the Ter macrodomain might be important for Topo IV targeting at *dif*.

Since the FtsK/Xer/*dif* system is dedicated to post-replicative events that are specific to a circular chromosome, it was tempting to postulate that the activity of Topo IV at *dif* is also dedicated to post-replicative decatenation events and is strictly required for circular chromosomes. To address this question, we used *E*. *coli* strains harboring linear chromosomes [34]. In this strain, expression of TelN from the N15 phage promotes linearization of the chromosome at the *tos* site inserted a 6kb away from *dif*. Indeed, chromosome linearization suppresses the phenotypes associated with *dif* deletion [34]. We analyzed cleavage at the *dif* site by Topo IV in the context of a linearized chromosome. Cleavage was completely abolished; showing that Topo IV activity at *dif* is not required on linear chromosomes. This effect was specific to the *dif* site, since cleavage at the 1.9Mb site remained unchanged after chromosome linearization (Fig 5F). We next assayed if the phenotypes associated with *matP* deletion, i.e., formation of elongated cells with non-partitioned nucleoids [26], depend on chromosome circularity. Strikingly, most of the phenotypes observed in the *matP* mutant were suppressed by linearization of the chromosome (Fig 5G). Interestingly, the frequency of cleavage at *dif* sites inserted far (300 kb) from the normal position of *dif* or in a plasmid were significantly reduced compared to the WT situation (S11 Fig) confirming that Topo IV cleavage at *dif* is specific to circular chromosomes.

## Discussion

### Specific Topo IV binding and cleavage sites on the chromosome

Whole genome analysis of Topo IV binding by ChIP-seq revealed approximately 10 Topo IV binding sites across the *E*. *coli* genome. Among them, only 5 sites were strongly enriched in every experiment and these were mapped to positions 1.25, 1.58 (*dif*), 1.85, 2.56 and 3.24 Mb. We did not identify any consensus sequence that could explain specific binding to these sites. Band shift experiments at the *dif* site and the 1.25 Mb site revealed that Topo IV binding is not sequence-dependent.

This led us to favor models involving exogenous local determinants for Topo IV binding as it is the case for the *dif* site in the presence of XerC. Because XerC is only known to bind to *dif*, we could speculate that other chromatin factors might be involved in Topo IV targeting. Topo IV and Fis binding sites [33] overlap more frequently than expected (Nust P value 10e-03 [32]. Topo IV and Fis binding sites overlap at the positions 1.25 and 2.56 Mb; it is therefore possible that Fis plays a role in defining some Topo IV binding sites. However our EMSA, cleavage and ChIP experiments did not show any cooperative binding of Topo IV with Fis. In spite of its co-localization with Topo IV, Fis does not contribute in defining Topo IV binding or cleavage sites. Nevertheless, the role of the chromatin in Topo IV localization was also illustrated by the strong negative correlation observed for the Topo IV and H-NS bound regions. H-NS rich regions were significantly less enriched for nonspecific Topo IV binding than the rest of the chromosome.

### Topo IV mediated DNA cleavage sites

We postulated that loci where Topo IV is catalytically-active could be identified by DNA cleavage mediated by the quinolone drug norfloxacin. We designed a new ChIP-seq strategy that consisted of capturing DNA-norfloxacin-Topo IV complexes. We called it NorflIP. Three independent experiments show that Topo IV was trapped to a large number of loci (300 to 600) with most of these loci observed in two out of three experiments. A hundred of these loci were identified in all three experiments. *Dif* presented a strong signal in the NorflIP as in the ChIP-seq but this is not the case for most of the other ChIP-seq peaks. NorflIP peaks presented a characteristic pattern suggesting that they are genuine DNA-norfloxacin-Topo IV complexes. Considering that norfloxacin does not alter Topo IV specificity, our results suggest that for Topo IV the genome is divided into five categories: i) Loci where Topo IV binds strongly but remains inactive for most of the cell cycle; ii) Loci where Topo IV is highly active but does not reside for very long time; iii) Loci where we observed both binding and activity (*dif* and 2.56 Mb); iv) regions where Topo IV interacts non-specifically with the DNA and where topological activity is not stimulated; v) regions where non-specific interactions are restricted (the Ter domain, chromatin rich regions (tsEPODs [35], H-NS rich regions). Detection of norfloxacin-mediated genomic cleavage by pulse field electrophoresis has previously revealed that when Topo IV is the only target of norfloxacin the average fragment size is 300–400 kb while it drops to 20 kb when Gyrase is the target [11]. This suggests that, for each cell, no more than 10 to 20 Topo IV cleavages are formed in 10 min of norfloxacin treatment. To fit this observation with our data, only a small fraction (10–20 out of 600) of the detected Topo IV cleavage sites would actually be used in each cell. This might explain why Topo IV cleavage sites were hardly distinguishable from background in the ChIP-seq assay (Fig 3). This is in good agreement with the estimation that the catalytic cycle only provokes a short pause (1.8 sec) in Topo IV dynamics [36]. The mechanism responsible for the choice of specific Topo IV cleavage sites is yet to be determined. As indicated by our findings that deletion of the cleavage site resulted in the formation of a new site or sites in the vicinity, cleavage is not directly sequence-related. We observed several biases that might be involved in determination of cleavage sites (GC di-nucleotide skew, GATC spacing, positioning near gene ends or intergenic regions, proximity with highly expressed genes and Fis binding regions). Interestingly inhibition of transcription with rifampicin inhibits Topo IV cleavage (Fig 3). This raises the possibility that transcription, that can be stochastic, may influence stochastic determination of Topo IV activity sites. The influence of transcription could be direct, if RNA polymerase pushes Topo IV to a suitable place, or indirect if the diffusion of topological constraints results in their accumulation near barriers imposed by gene expression [37, 38]. This accumulation could then, in turn, signal for the recruitment of Topo IV.

### Replication influences Topo IV binding and activity

Synchronization experiments revealed that, like Topo IV binding at specific sites, Topo IV cleavage activity is enhanced by chromosome replication. Enrichment was the highest in late S phase or G2 phase; it seems to persist after the passage of the replication fork at a defined locus. Enrichment in asynchronous cultures was significantly reduced compared to S40 or G2 synchronized cultures suggesting that Topo IV is not bound to the chromosome for the entire cell cycle. Unfortunately our experiments did not have the time resolution to determine at what point of the cell cycle Topo IV leaves the chromosome and if it would leave the chromosome during a regular cell cycle. The role of DNA replication of Topo IV dynamics has recently been observed by a very different approach [36]. The authors propose that Topo IV accumulates in the *oriC* proximal part of the chromosome in a MukB and DNA replication dependent process. These observations are in good agreement with our data and suggest that Topo IV is loaded on DNA at the time of replication, accumulate towards the origin of replication and remains bound to the DNA until a yet unidentified event triggers its release. Formation of positive supercoils and precatenanes ahead and behind of the replication forks respectively, could be the reason for Topo IV recruitment. One could hypothesize that MukB is used as a DNA topology sensor that is responsible for redistribution of Topo IV. However we only detected a modest effect of *mukB* deletion on Topo IV cleavage at *dif* (Fig 5). Putative events responsible for Topo IV release could be, among others, complete decatenation of the chromosome, SNAPs release, or stripping by other proteins such as FtsK.

### Non-specific Topo IV binding

Non-specific Topo IV binding presents a very peculiar pattern; it is significantly higher in the *oriC* proximal 3Mb than in the 1.6Mb surrounding *dif*. This pattern is not simply explained by the influence of replication (S3 Fig). Interestingly, ChIP-seq and ChIP-on-Chip experiments have already revealed a similar bias for DNA gyrase [12] and SeqA [39]. The CbpA protein has been shown to present an inverse binding bias [40], with enrichment in the terminal region and a reduction in the *oriC* proximal domain. The HU regulon has also presented a similar bias [41]. The terminus domain defined by these biases always comprises the Ter macrodomain but it extends frequently beyond the extreme *matS* sites. The role of MatP in the definition of these biases has not yet been tested. The group of G. Mushelishvili proposed a topological model to interpret the DNA gyrase and HU regulon biases, suggesting that HU coordinates the global genomic supercoiling by regulating the spatial distribution of RNA polymerase in the nucleoid [41]. Topo IV could benefit from such a supercoiling gradient to load on the chromosome. Interestingly, the strongest Topo IV binding and cleavage sites are localized inside the Terminus depleted domain. One possibility could be that these sites minimize Topo IV binding to adjacent nonspecific sequences. Alternatively one can propose that a regional reduction of non-specific binding creates a selective advantage for optimal loading on to specific sites.

### *Dif* and the control of decatenation

*Dif* was the strongest Topo IV cleavage site detected by NorflIP, it was also detected in the ChIP-seq assays. We have used Southern blot to analyze the determinants involved in this activity. The binding of XerC on the xerC box of *dif* and the region downstream of the *xerC* box are essential. *In vitro*, XerC also strongly favors binding of Topo IV at *dif*. Interestingly XerD and the *xerD* box did not improve Topo IV binding or cleavage. We propose that XerC works as a scaffold for Topo IV, simultaneously stimulating its binding and its activity. Topo IV activity at *dif* is also dependent on the circularity of the chromosome, suggesting that when topological constraints can be evacuated through chromosome ends, Topoisomerase IV does not catalyze strand passage at *dif*. This suggests that topological complexity is directly responsible for Topo IV activity. Topo IV cleavage activity at *dif* is not influenced by SeqA or FtsK, which are two known Topo IV partners. Interestingly, *mukB* and *matP* deletion mutants slightly reduced this activity. The synergistic effect observed when a *matP* deletion is combined with a *parEts* mutation suggests that MatP indeed influences Topo IV activity. The phenotypes of the *matP* mutant are rescued by the linearization of the chromosome. A similar rescue has been observed for the *dif* mutant [34]. Therefore it is likely that a significant part of the problems that cells encounter in the absence of *matP* corresponds to failure in chromosome topology management, either decatenation or chromosome dimer resolution [25]. In conclusion, we propose that genomic regulation of Topo IV consists of: (1) Topo IV loading during replication, (2) Topo IV binding to specific sites that may serve as reservoirs, (3) Topo IV activation to remove precatenanes or positive supercoils in a dozen of stochastically chosen loci (4) XerC and MatP ensuring the loading of Topo IV at the *dif* site for faithful decatenation of fully replicated chromosomes.

## Materials and Methods

### ChIP-seq assay

ParE-flag and ParC-flag C-terminus fusions were constructed by lambda red recombination [42]. Cultures were grown in LB or Minimal medium A supplemented with succinate (0.2%) and casamino acids (0.2%). Cells were fixed with fresh Formaldehyde (final concentration 1%) at an OD_600nm_ 0.2–0.4. Sonication was performed with a Bioruptor Pro (Diagenode). Immunoprecipitations were performed as previously described ^26^. Libraries were prepared according to Illumina's instructions accompanying the DNA Sample Kit (FC-104-5001). Briefly, DNA was end-repaired using a combination of T4 DNA polymerase, *E*. *coli* DNA Pol I large fragment (Klenow polymerase) and T4 polynucleotide kinase. The blunt, phosphorylated ends were treated with Klenow fragment (3’ to 5’ exo minus) and dATP to yield a protruding 3- 'A' base for ligation of Illumina's adapters which have a single 'T' base overhang at the 3’ end. After adapter ligation DNA was PCR amplified with Illumina primers for 15 cycles and library fragments of ~250 bp (insert plus adaptor and PCR primer sequences) were band isolated from an agarose gel. The purified DNA was captured on an Illumina flow cell for cluster generation. Libraries were sequenced on the Genome Analyzer following the manufacturer's protocols.

### Norflip assay

Norfloxacin (final concentration 2μM) was added to the cultures at OD_600nm_ 0.2 LB for 10 min before harvesting. Sonication and immunoprecipitation were performed as described for the ChIP-seq assay.

### Analysis of sequencing results

Sequencing results were processed by the IMAGIF facility. Base calls were performed using CASAVA version 1.8.2. ChIP-seq and NorflIP reads were aligned to the E. coli NC_000913 genome using BWA 0.6.2. A custom made pipeline for the analysis of sequencing data was developed with Matlab (available on request). Briefly, the number of reads for the input and IP data was smoothed over a 200bp window. Forward and reverse signals were added, reads were normalized to the total number of reads in each experiment, strong non-specific signals observed in unrelated experiments were removed, data were exported to the UCSC genome browser for visualization and comparisons. The strongest peaks observed with NorflIP experiments (*dif* and 1.9 Mb) present a characteristic shape (S6 Fig) that allows the automatic detection of lower amplitude peaks but preserves the characteristic shape. We measured Pearson correlation coefficient with the *dif* and the 1.9 Mb site for 600bp sliding windows over the entire genome. Peaks with a Pearson correlation above 0.72 were considered as putative Topo IV cleavage sites. Sequencing data are available on the GEO Repository (http://www.ncbi.nlm.nih.gov/geo/)with the accession number GSE75641. Data were plotted with the Circos tool [43] and UCSC Archaeal Genome Browser [44].

### Southern blot

Cleavage of DNA by Topo IV in the presence of Norfloxacin was monitored by Southern blot as previously described [10]. DNA was extracted from E. coli culture grown in minimal medium supplemented with glucose 0.2% and casaminoacids 0.2%. Norfloxacin (final concentration 10μM) was added to the cultures at OD 0.2 for 10 min before harvesting. DNA was transferred by neutral blotting on nitrocellulose membranes. For synchronization experiments a flash freeze step in liquid nitrogen is included before harvesting. Quantification was performed with Image J software.

### EMSA

Experiments were conducted using Cy3-coupled probes harboring the *dif* site and a Cy5-coupled dye as control. Reactions were carried out in EMSA reaction buffer (1mM spermidine, 30mM potassium glutamate, 10mM DTT, 6mM magnesium chloride, 10% glycerol, pH 7.4). Reactions were incubated for 15 min at RT, loaded on 4% native PAGE gel at 25 volts and then run at 125 volts for 2 hours. Gels were then visualized using a Typhoon FLA 5000 scanner (GE healthcare Life Science). EMSA of plasmids were performed with unlabeled supercoiled plasmid in the same reaction buffer. Electrophoresis was performed in a 0.8% agarose gel in 0.5x TAE buffer at 4°C for 80 min at 150V. DNA labeling was performed with SYBR green.

## Supporting Information

S1 Fig **A)** Measure of the colony formation unit (CFU) of the WT, *nalR*, ParC-Flag, ParC-Flag *nalR* and ParE-Flag *nalR* strains. Culture were grown until OD 0.2 and treated for 40 minutes with norfloxacin 2μM and plated on LB plates. **B)** Measure of the growth rate of the *nalR*, ParC-Flag *nalR* and ParE-Flag *nalR* strains. **C)** Southern blot analysis of Topo IV mediated cleavage in the presence of norfloxacin at the 1.9 Mb site in the WT, *nalR* and ParC-Flag *nalR* and ParE-Flag *nalR* strains.
(PDF)

S2 Fig Genome browser magnifications illustrating common non-specific signal observed over rRNA operons, tRNA and IS sequences. ParE-Flag ChIP-seq is represented in red, MatP-Flag ChIP-seq is represented in blue, Mock IP with a strain that did not contain Flag tagged proteins is represented in black. Genes, ribosomal operons and tRNA are represented below ChIPseq signals
(PDF)

S3 Fig **A)** Analysis of the Topo IV nonspecific binding. Normalized enrichment (Average number of reads in a 1kb sliding window divided by the total amount of reads) of each flag immuno-precipitation experiment was plotted as a function of the genomic position. Left panel a 100 kb region near *oriC* (positions 4.26 to 4.36 Mb) is represented. Right panel a 100 kb region around *dif* (positions 1.55 to 1.65 Mb) is represented. **B)** Scatter plot of the average GC content according to *parC-flag* IP/Input. 60 kb sliding windows were used for GC content and IP/Input. **C)** Average IP/Input values were normalized for GC content. **D)** Null model I, a Topo IV comet follows replication forks. Illustration of the Topo IV binding kinetics under null model I described in S1 Text. The x axis in the plots represents the chromosome coordinate s, going between 0 (ori) and L (ter). The y axis represents cell cycle time. The shaded areas are the positions of the Topo IV comets (also sketched as red lines on a circular representation of the chromosome), and the numbers represent the number of bound regions per replichore. **Left panel:** case of non-overlapping rounds. **Right panel:** case of overlapping rounds, in the case where the B period starts after the termination of replication within the same cell cycle. **E)** Topo IV binding bias, shown by the specific Input/IP values (each normalized by total reads). This bias is not compatible with a model where Topo IV binding follows replication and persists for a characteristic period of time (purple trace).
(PDF)

S4 Fig Flow cytometry analysis of the synchronization experiment. Samples were fixed in ethanol at different time points: after 1h30 at 40°C (G1), 20 min after downshift to 30°C (S20), 40 min after downshift to 30°C (S40), 60 min after downshift to 30°C (G2) and in stationary phase.
(PDF)

S5 Fig **A)** Genome browser magnifications illustrating common non specific signal observed over rRNA operon, IS sequences in the NorflIP and ChIP-seq experiments. ParE-Flag NorflIP is represented in purple, MatP-Flag ChIP-seq is represented in blue, Mock IP with a strain that did not contained Flag tagged proteins is represented in black. Genomic localization are the same as in S2 Fig **B)** Southern blot cleavage assays performed in WT and *nalR* strains at the *insH* locus, ribosomal operon A and ribosomal operon B. TopoIV did not present any cleavage in this regions confirming the artefactual nature of the corresponding signals in the NorflIP experiments. Arrows indicated the position on the corresponding bottom map.
(PDF)

S6 Fig **A)** Snapshots of the ChIP-seq and NorflIP experiments at the position 1.85 and 1.92 Mb. Topo IV binding to position 1.85 Mb was only revealed by the ChIP-seq experiment in the presence of formaldehyde. Topo IV cleavage at position 1.92 Mb was only revealed by the NorflIP experiment. NorflIP peaks present a characteristic shape illustrated on the 1.92Mb with a large 200 bp empty region in between the forward and reverse signal (arrow). **B)** Snapshot of the ChIP-seq and NorflIP experiments at the dif position. Topo IV binding (ChIP-seq) and cleavage (NorflIP) were detected at the *dif* position. **C)** Description of the NorflIP peak calling procedure. Forward and reverse reads from the Flag immunoprecipitation were smoothed over 200 bp, and then subtracted from each other. The *dif* and 1.9Mb signals observed on a 2kb window were used as a probe to test the entire genome with 100 bp sliding intervals. Pearson coefficient between the *dif* and 1.9 Mb signals and each interval were measured. Pearson coefficients above 0.72 were considered as putative Topo IV peaks. The initial list of Topo IV sites (S1 Table) corresponds to sites presenting a Pearson correlation above 0.72 in comparison with *dif* and 1.9Mb. IP/input ratio was measured. 172 peaks with Pearson coefficient above 0.72 and an IP/input ratio >2 were manually validated as Topo IV sites (S1 Table). **D)** Analysis of reads orientation in the NorflIP experiment at position 0.2Mb. Forward and reverse read peaks are about 200 bp large, a 100 nucleotides gap is observed in between the peaks. For the analysis of Topo IV cleavage site distribution we estimated that the center of the 100 nucleotides gap corresponds to the position of Topo IV cleavage.
(PDF)

S7 Fig Measure of the distance between two adjacent Topo IV cleavage sites in the *dif* region **(A)** and the region containing *oriC* and SNAP2 **(B).** For this analysis the 571 Topo IV cleavage sites observed in the 3 experiments were pooled. **C)** Distribution of the Topo IV cleavages inside genes and intergenic regions. The gene sizes were normalized to 1. **D)** RSAT analysis of the NorflIP peak calling results (http://www.rsat.eu/; Thomas-Chollier M, Defrance M, Medina-Rivera A, Sand O, Herrmann C, Thieffry D, van Helden J. (2011) RSAT 2011: regulatory sequence analysis tools. Nucleic Acids Res. 2011 Jul;39. Analysis of the dinucleotide bias in 172 manually validated NorflIP Topo IV cleavage sites. In average GC dinucleotides are enriched near the middle of the ChIP signal. **E)** GATC spacing around Topo IV peaks detected with the NorflIP experiment. Average distances between two consecutive GATC are measured around (+/- 20 GATC sites) 172 validated Topo IV cleavage sites and 172 random sequences.
(PDF)

S8 Fig **A)** Box plot of the distribution of distance between TopoIV cleavages and the closest highly expressed transcription unit (T.U.). For this analysis the 571 Topo IV cleavage sites observed in the 3 experiments were pooled. T.U. expression was determined by RNAseq. An arbitrary threshold was set to 500 reads, it corresponds to the 10% of the T.U. the most expressed. The distribution of a random set of cleavage sites was used as control. The two distributions are statistically different according to Anova test. The median distance is 8.5 kb for the TopoIV cleavage set and 12.3 kb for the random set. **B)** Genome browser zoom on the region 1.92 Mb were TopoIV cleavages were observed in a region with a number of highly expressed T.U. **C)** Distribution of 458 Topo IV cleavages (black) and random sites (grey) in between two consecutive highly expressed T. U. Topo IV cleavages are slightly more frequent near the TU than in the middle of the region.
(PDF)

S9 Fig **A)** Distribution of ParE-Flag 1 ChIP-seq enrichment in the region overlapping or not a H-NS binding site. **B)** Box plot of the distribution of GC% in the regions depleted for Topo IV (IP/input <0.6) or enriched for Topo IV (IP/input >1.2) or enriched for H-NS. **C)** Distribution of the GC% in 172 validated Topo IV cleavage sites as function of NorflIP IP/input signal. **D)** Measure of the GC% in the 172 validated cleavage sites. GC % was measured in sliding windows of 20 bp and color coded.
(PDF)

S10 Fig **A)** Analysis of the robustness of the Topo IV-XerC-*dif* complex in the presence of increasing amounts of XerD protein. EMSA were performed with prebound Topo IV and XerC on *dif* and subsequent addition of XerD for 10 minutes before loading on the gel. **B)** Analysis of Topo IV binding to negatively supercoiled plasmid by EMSA on agarose gel. Topo IV from 10, 50, 100, 200 nM was added to the pFC24 (dif) plasmid in the presence of XerCD (25 or 50 nM).
(PDF)

S11 Fig **A)** Southern Blot analysis of Topo IV cleavage in the *nalR* strain at *dif* and an ectopic *dif* site located at 1.3Mb on the genomic map. **B)** Southern Blot analysis of Topo IV cleavage on a plasmid (pFC25) carrying the *dif* region (10 kb around *dif*) + or–*dif*
(PDF)

S1 Table **Sheet 1)** Validated ChIP-seq sites. **Sheet 2)** NorflIP sites observed in the ParC-Flag 1 NorflIP, ParE-Flag NorflIP and ParC-Flag 2 NorflIP. **Sheet 3)** Common NorflIP sites for the different experiments. **Sheet 4)** Manually Validated Topo IV cleavages.
(XLSX)

S1 Text Model to test the correlation between TopoIV binding and the progression of replication. To test if ParC and ParE ChIP-seq biases were related to chromosome replication we constructed *in silico* models The result of this null model is that in all cases (overlapping or non-overlapping rounds) the observed mean occupancy should follow the dosage. Hence the occupancy gap observed in S3E Fig in the Ter region (when occupancy is normalized by dosage) has to be interpreted as a sign that this model does not apply, at least in this region.
(DOCX)
