# Supplementary figures and images for: Mapping Topoisomerase IV Binding and Activity Sites on the E. coli Genome
Source: PLoS Genet. 2016 May 12;12(5):e1006025. doi: 10.1371/journal.pgen.1006025 (PMC4865107; doi:10.1371/journal.pgen.1006025)

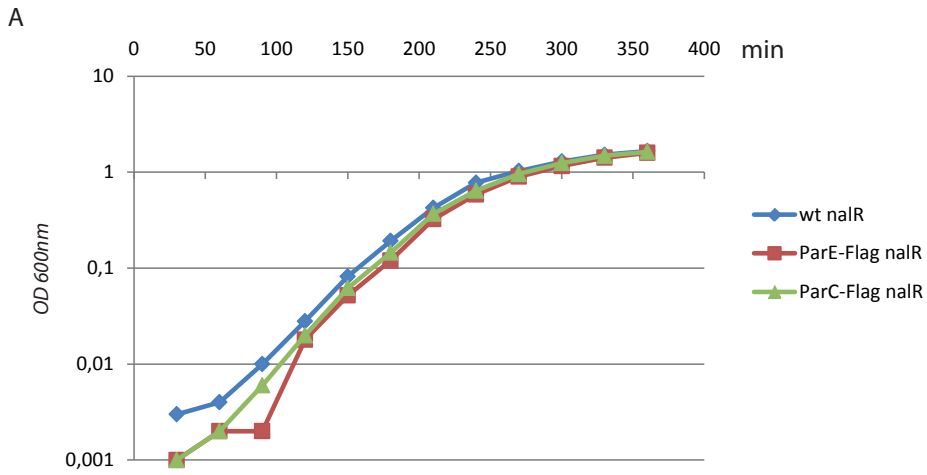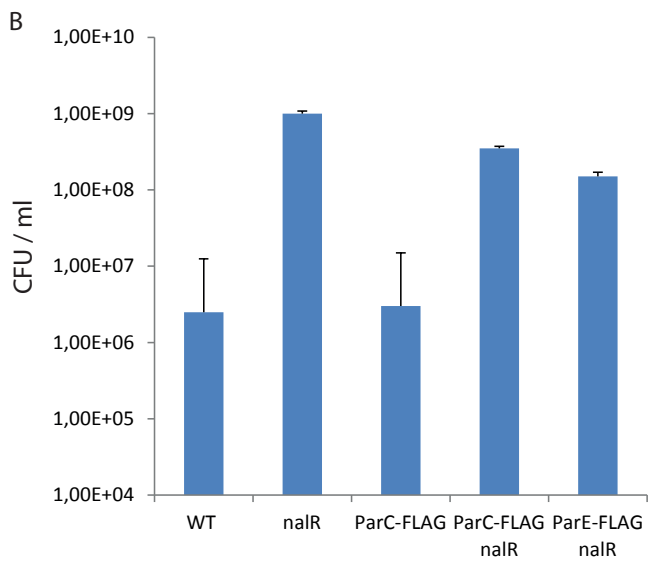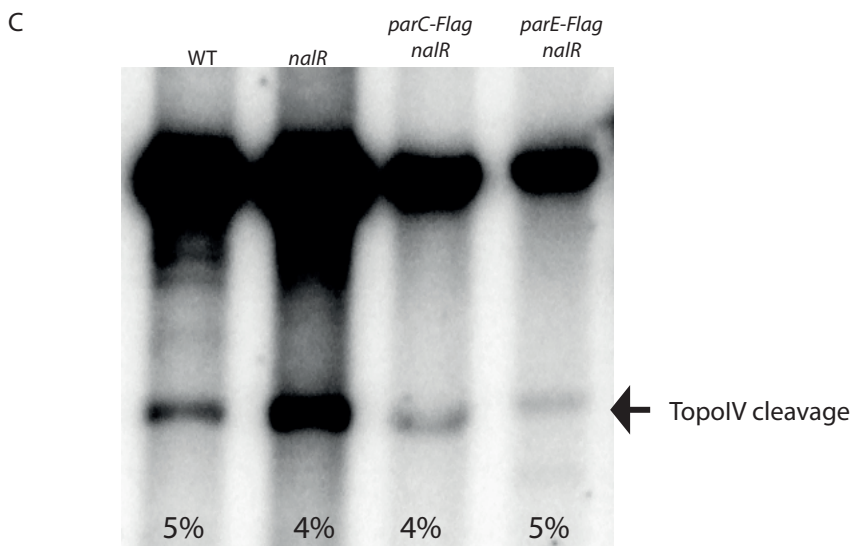

S1 Fig.

Supplement: S1 Fig — A) Measure of the colony formation unit (CFU) of the WT, nalR, ParC-Flag, ParC-Flag nalR and ParE-Flag nalR strains. Culture were grown until OD 0.2 and treated for 40 minutes with norfloxacin 2μM and plated on LB plates. B) Measure of the growth rate of the nalR, ParC-Flag nalR and ParE-Flag nalR strains. C) Southern blot analysis of Topo IV mediated cleavage in the presence of norfloxacin at the 1.9 Mb site in the WT, nalR and ParC-Flag nalR and ParE-Flag nalR strains. (PDF) [file pgen.1006025.s001.pdf]

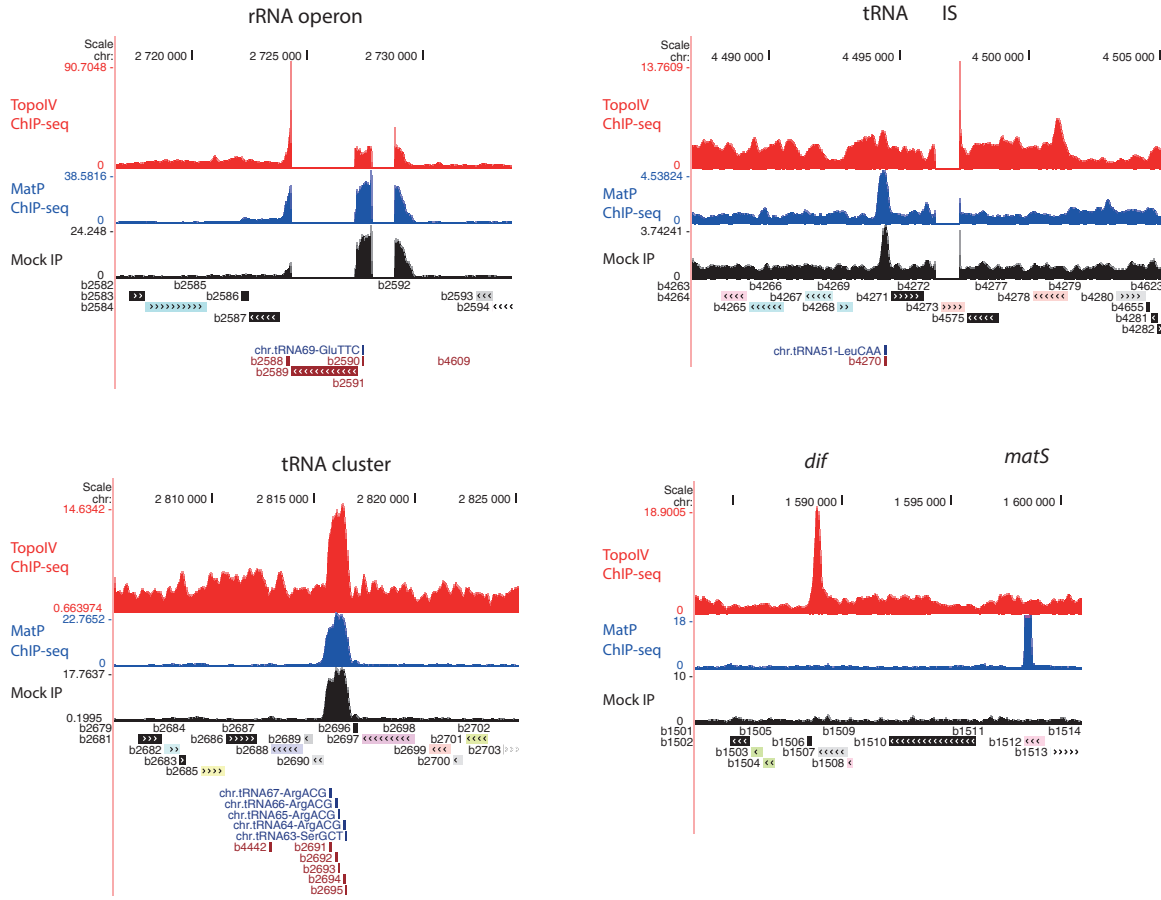

S2 Fig.

Supplement: S2 Fig — ParE-Flag ChIP-seq is represented in red, MatP-Flag ChIP-seq is represented in blue, Mock IP with a strain that did not contain Flag tagged proteins is represented in black. Genes, ribosomal operons and tRNA are represented below ChIPseq signals (PDF) [file pgen.1006025.s002.pdf]

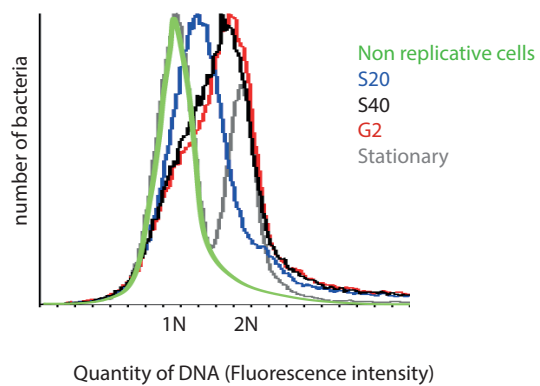

S4 Fig.

Supplement: S4 Fig — Samples were fixed in ethanol at different time points: after 1h30 at 40°C (G1), 20 min after downshift to 30°C (S20), 40 min after downshift to 30°C (S40), 60 min after downshift to 30°C (G2) and in stationary phase. (PDF) [file pgen.1006025.s004.pdf]

A

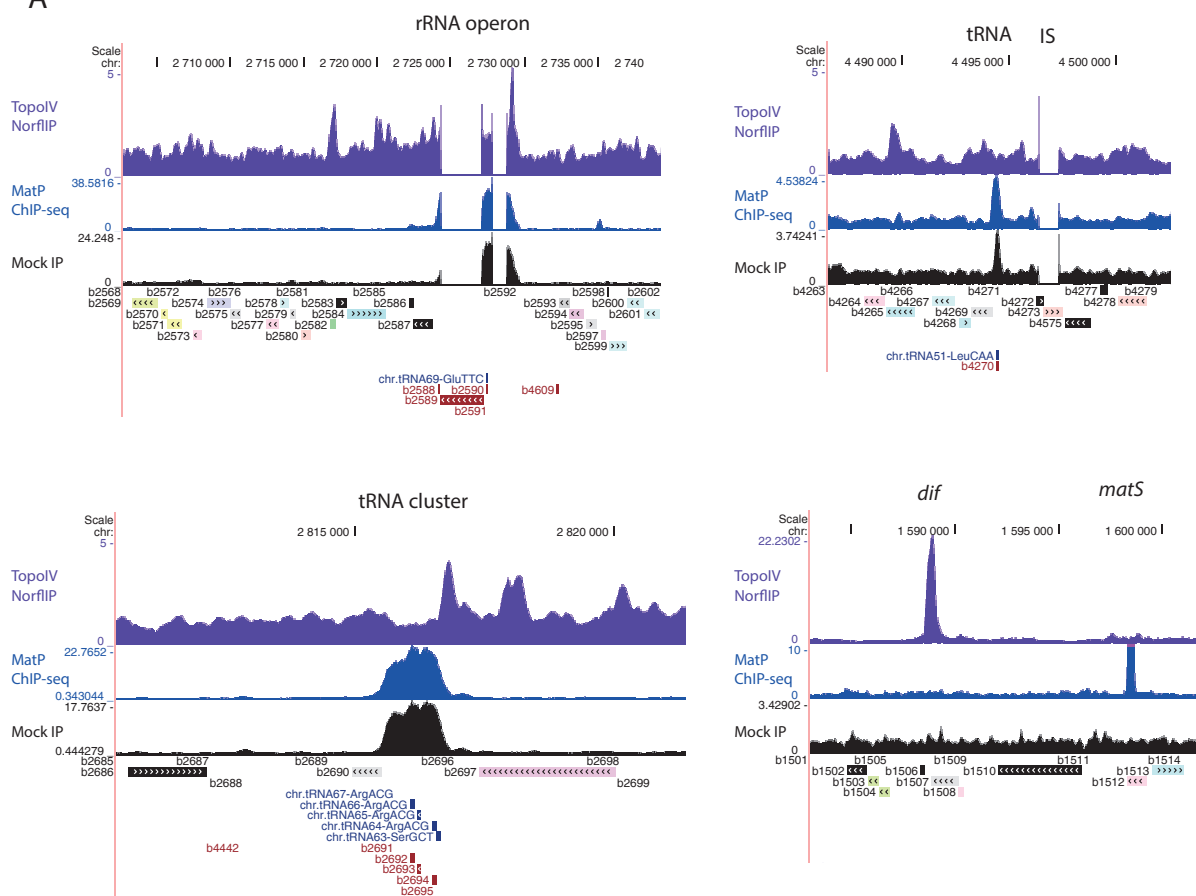

B

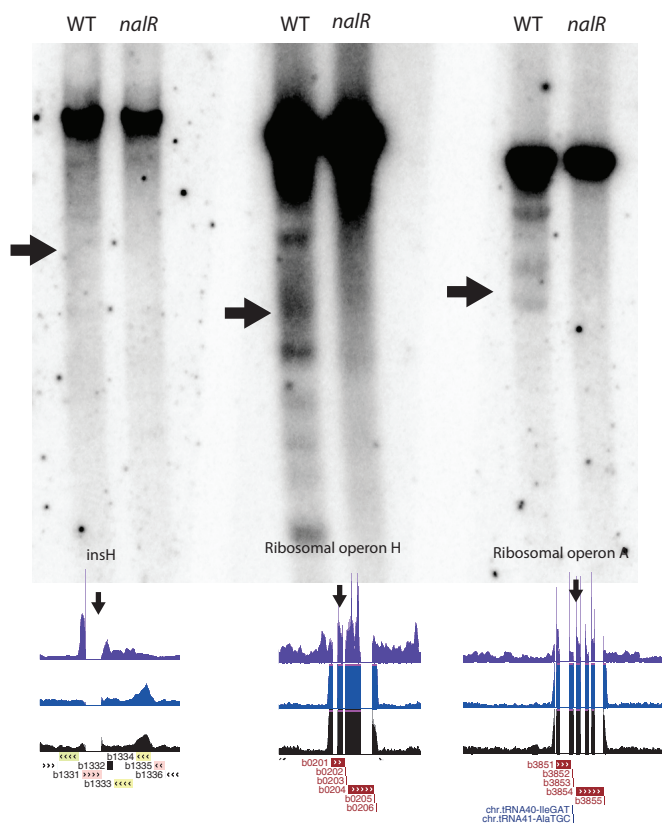

Supplement: S5 Fig — A) Genome browser magnifications illustrating common non specific signal observed over rRNA operon, IS sequences in the NorflIP and ChIP-seq experiments. ParE-Flag NorflIP is represented in purple, MatP-Flag ChIP-seq is represented in blue, Mock IP with a strain that did not contained Flag tagged proteins is represented in black. Genomic localization are the same as in S2 Fig B) Southern blot cleavage assays performed in WT and nalR strains at the insH locus, ribosomal operon A and ribosomal operon B. TopoIV did not present any cleavage in this regions confirming the artefactual nature of the corresponding signals in the NorflIP experiments. Arrows indicated the position on the corresponding bottom map. (PDF) [file pgen.1006025.s005.pdf]

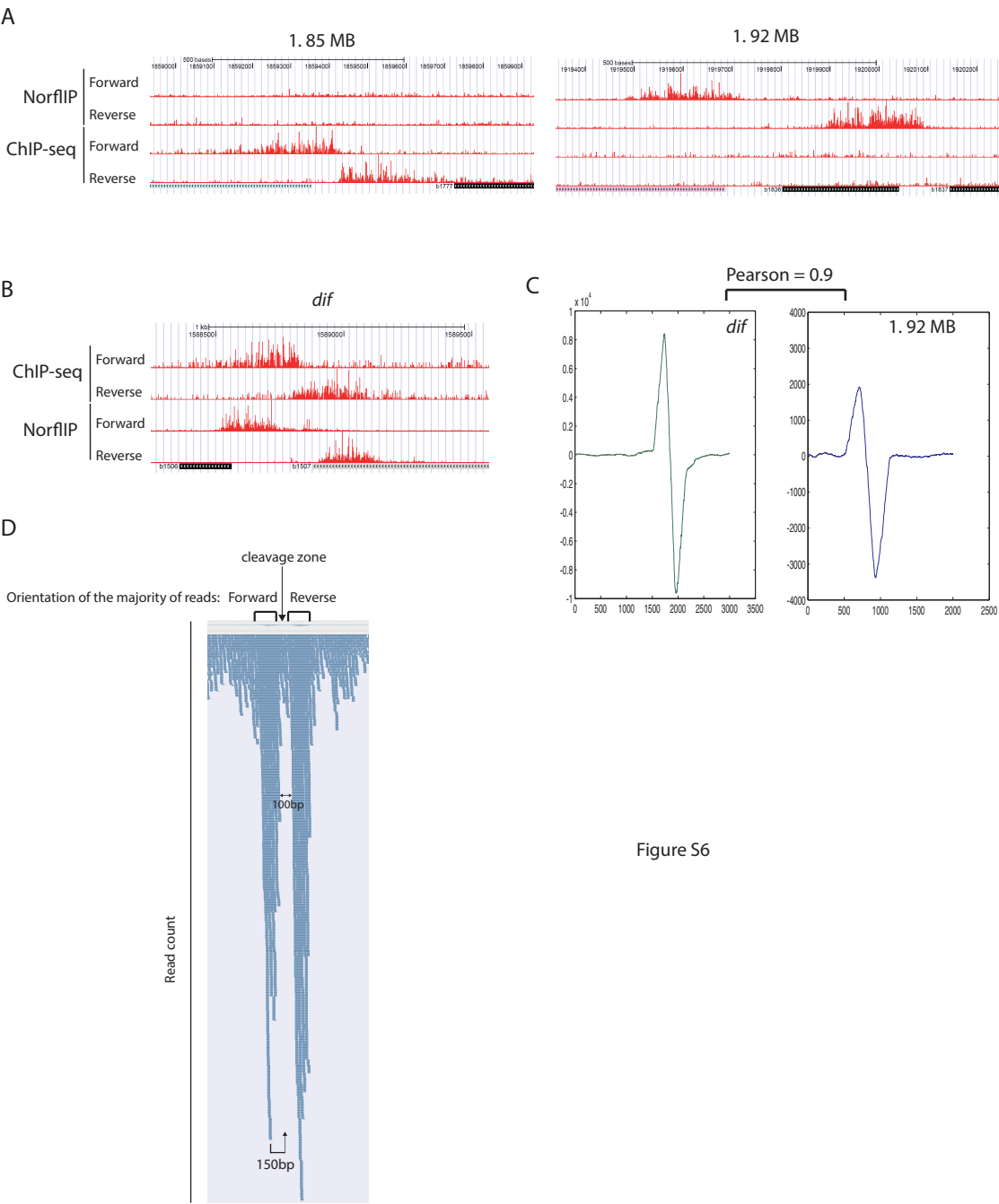

Figure S6

Supplement: S6 Fig — A) Snapshots of the ChIP-seq and NorflIP experiments at the position 1.85 and 1.92 Mb. Topo IV binding to position 1.85 Mb was only revealed by the ChIP-seq experiment in the presence of formaldehyde. Topo IV cleavage at position 1.92 Mb was only revealed by the NorflIP experiment. NorflIP peaks present a characteristic shape illustrated on the 1.92Mb with a large 200 bp empty region in between the forward and reverse signal (arrow). B) Snapshot of the ChIP-seq and NorflIP experiments at the dif position. Topo IV binding (ChIP-seq) and cleavage (NorflIP) were detected at the dif position. C) Description of the NorflIP peak calling procedure. Forward and reverse reads from the Flag immunoprecipitation were smoothed over 200 bp, and then subtracted from each other. The dif and 1.9Mb signals observed on a 2kb window were used as a probe to test the entire genome with 100 bp sliding intervals. Pearson coefficient between the dif and 1.9 Mb signals and each interval were measured. Pearson coefficients above 0.72 were considered as putative Topo IV peaks. The initial list of Topo IV sites (S1 Table) corresponds to sites presenting a Pearson correlation above 0.72 in comparison with dif and 1.9Mb. IP/input ratio was measured. 172 peaks with Pearson coefficient above 0.72 and an IP/input ratio >2 were manually validated as Topo IV sites (S1 Table). D) Analysis of reads orientation in the NorflIP experiment at position 0.2Mb. Forward and reverse read peaks are about 200 bp large, a 100 nucleotides gap is observed in between the peaks. For the analysis of Topo IV cleavage site distribution we estimated that the center of the 100 nucleotides gap corresponds to the position of Topo IV cleavage. (PDF) [file pgen.1006025.s006.pdf]

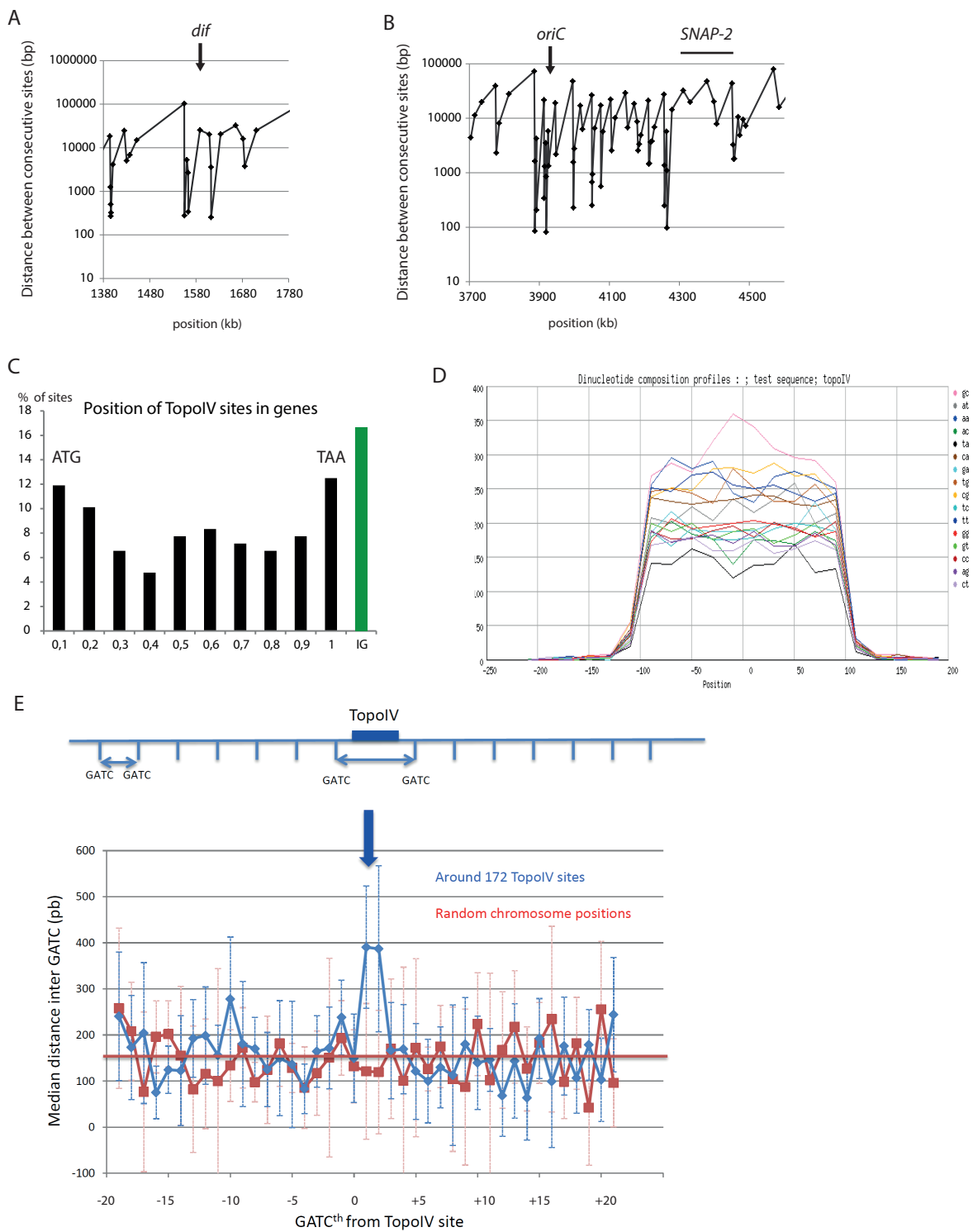

S7 Fig.

Supplement: S7 Fig — Measure of the distance between two adjacent Topo IV cleavage sites in the dif region (A) and the region containing oriC and SNAP2 (B). For this analysis the 571 Topo IV cleavage sites observed in the 3 experiments were pooled. C) Distribution of the Topo IV cleavages inside genes and intergenic regions. The gene sizes were normalized to 1. D) RSAT analysis of the NorflIP peak calling results (http://www.rsat.eu/; Thomas-Chollier M, Defrance M, Medina-Rivera A, Sand O, Herrmann C, Thieffry D, van Helden J. (2011) RSAT 2011: regulatory sequence analysis tools. Nucleic Acids Res. 2011 Jul;39. Analysis of the dinucleotide bias in 172 manually validated NorflIP Topo IV cleavage sites. In average GC dinucleotides are enriched near the middle of the ChIP signal. E) GATC spacing around Topo IV peaks detected with the NorflIP experiment. Average distances between two consecutive GATC are measured around (+/- 20 GATC sites) 172 validated Topo IV cleavage sites and 172 random sequences. (PDF) [file pgen.1006025.s007.pdf]

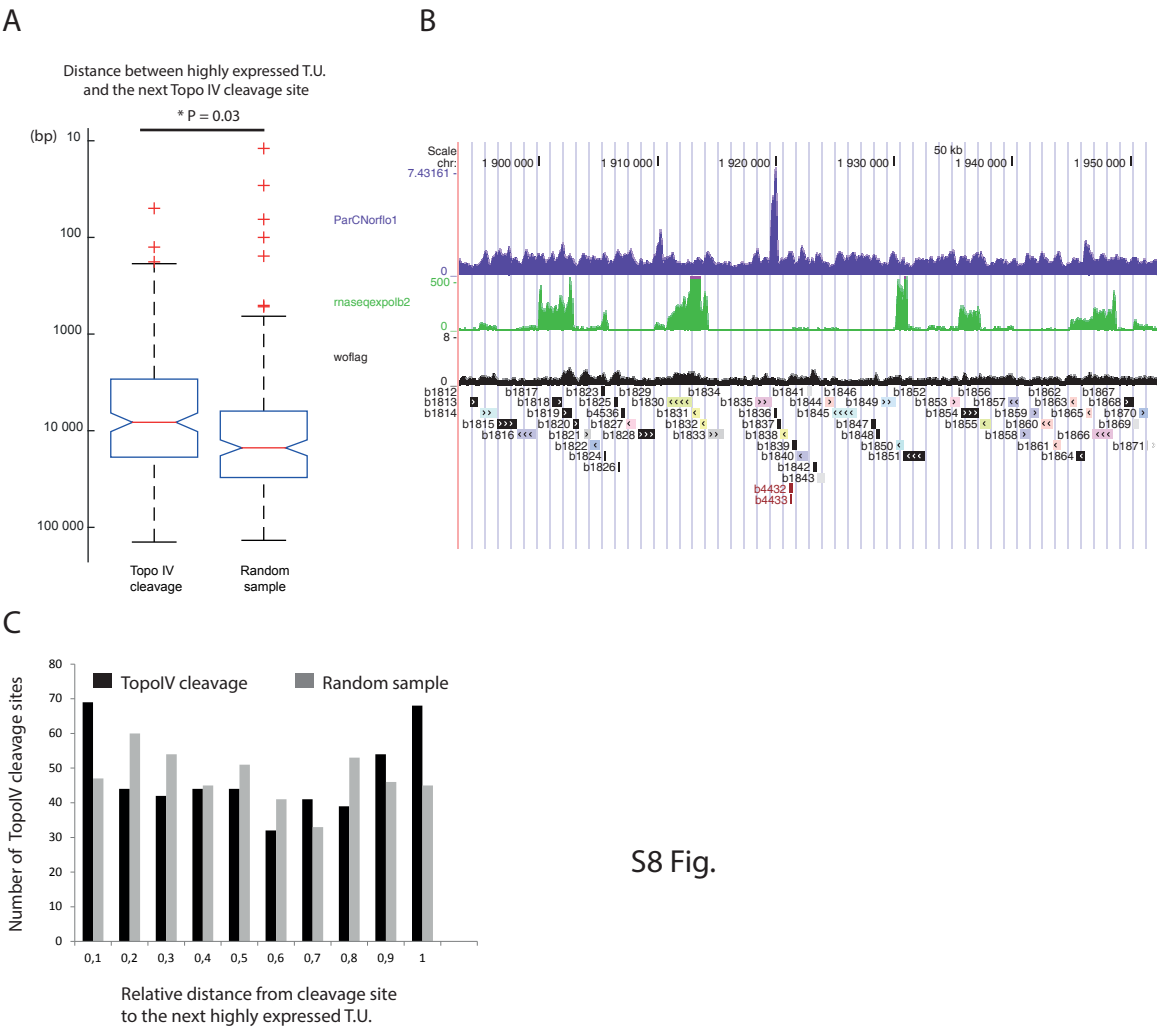

S8 Fig.

Supplement: S8 Fig — A) Box plot of the distribution of distance between TopoIV cleavages and the closest highly expressed transcription unit (T.U.). For this analysis the 571 Topo IV cleavage sites observed in the 3 experiments were pooled. T.U. expression was determined by RNAseq. An arbitrary threshold was set to 500 reads, it corresponds to the 10% of the T.U. the most expressed. The distribution of a random set of cleavage sites was used as control. The two distributions are statistically different according to Anova test. The median distance is 8.5 kb for the TopoIV cleavage set and 12.3 kb for the random set. B) Genome browser zoom on the region 1.92 Mb were TopoIV cleavages were observed in a region with a number of highly expressed T.U. C) Distribution of 458 Topo IV cleavages (black) and random sites (grey) in between two consecutive highly expressed T. U. Topo IV cleavages are slightly more frequent near the TU than in the middle of the region. (PDF) [file pgen.1006025.s008.pdf]

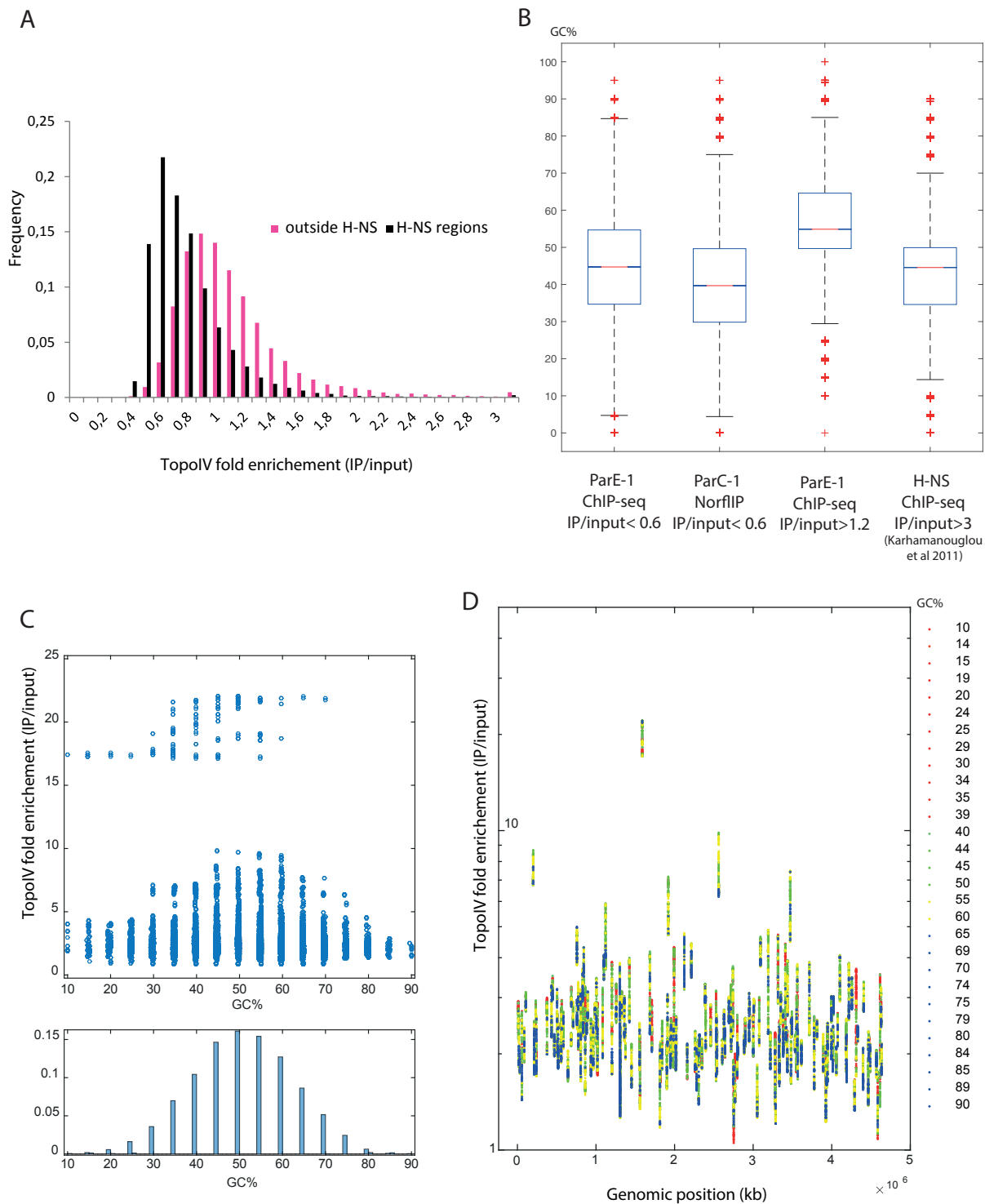

S9 Fig.

Supplement: S9 Fig — A) Distribution of ParE-Flag 1 ChIP-seq enrichment in the region overlapping or not a H-NS binding site. B) Box plot of the distribution of GC% in the regions depleted for Topo IV (IP/input <0.6) or enriched for Topo IV (IP/input >1.2) or enriched for H-NS. C) Distribution of the GC% in 172 validated Topo IV cleavage sites as function of NorflIP IP/input signal. D) Measure of the GC% in the 172 validated cleavage sites. GC % was measured in sliding windows of 20 bp and color coded. (PDF) [file pgen.1006025.s009.pdf]

A

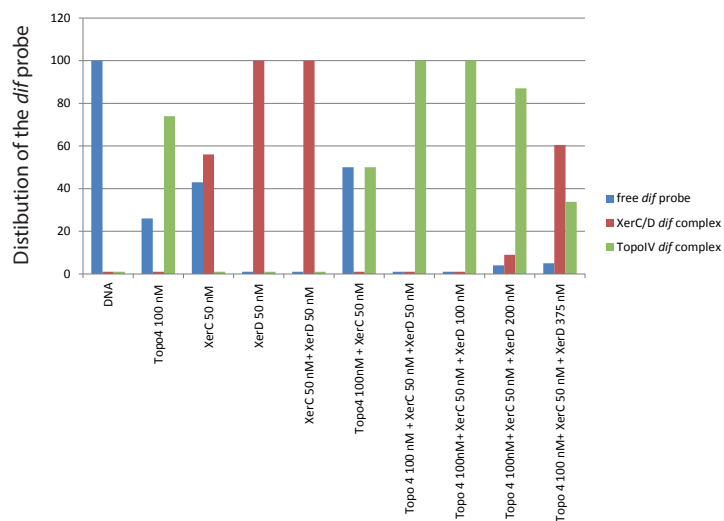

B

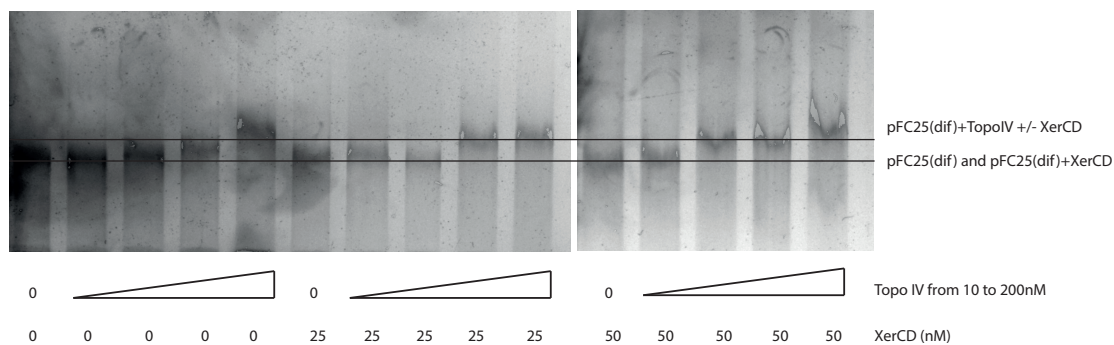

Supplementary Figure S10

Supplement: S10 Fig — A) Analysis of the robustness of the Topo IV-XerC-dif complex in the presence of increasing amounts of XerD protein. EMSA were performed with prebound Topo IV and XerC on dif and subsequent addition of XerD for 10 minutes before loading on the gel. B) Analysis of Topo IV binding to negatively supercoiled plasmid by EMSA on agarose gel. Topo IV from 10, 50, 100, 200 nM was added to the pFC24 (dif) plasmid in the presence of XerCD (25 or 50 nM). (PDF) [file pgen.1006025.s010.pdf]

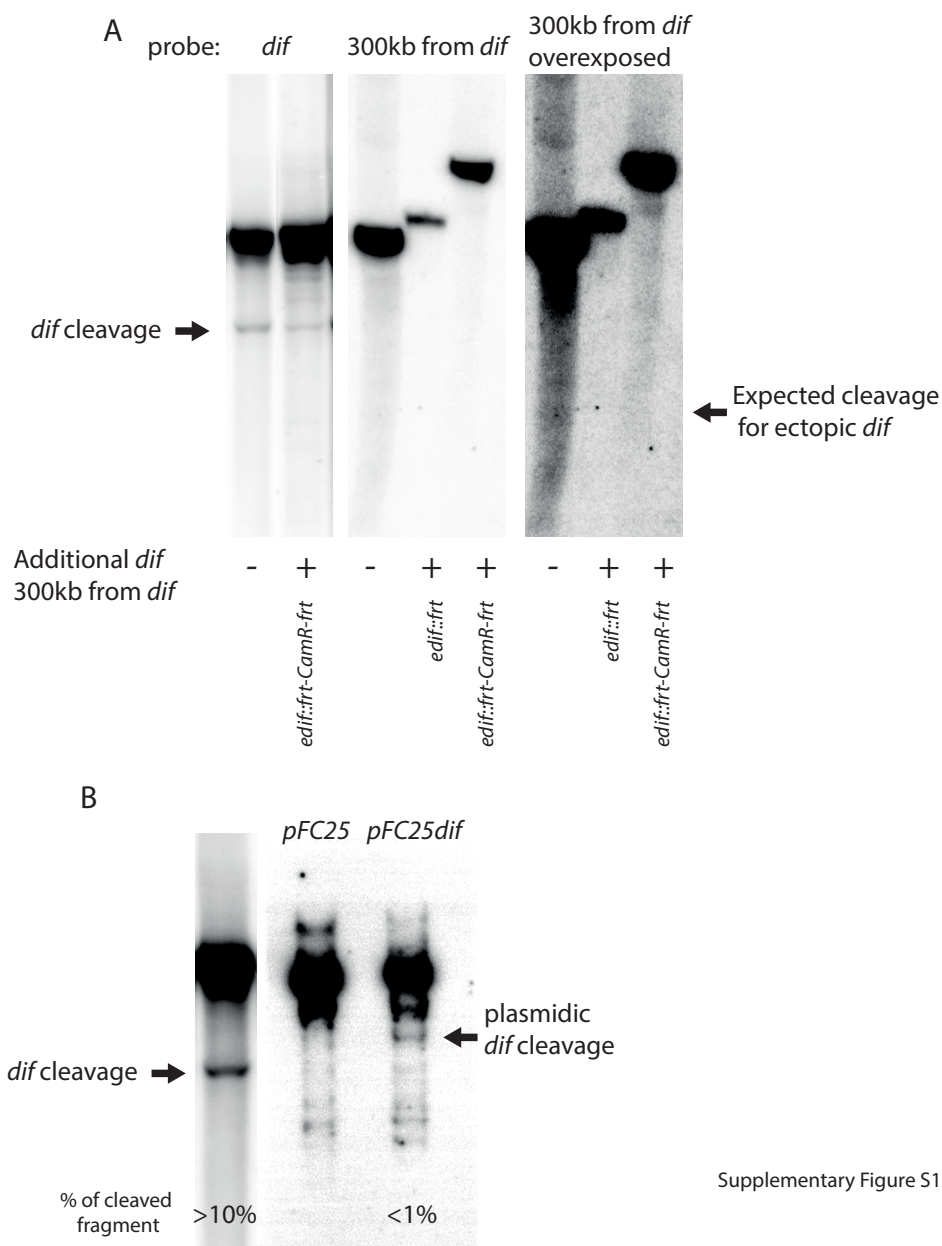

Supplementary Figure S11

Supplement: S11 Fig — A) Southern Blot analysis of Topo IV cleavage in the nalR strain at dif and an ectopic dif site located at 1.3Mb on the genomic map. B) Southern Blot analysis of Topo IV cleavage on a plasmid (pFC25) carrying the dif region (10 kb around dif) + or–dif (PDF) [file pgen.1006025.s011.pdf]
