# Supplementary material for: Mapping Topoisomerase IV Binding and Activity Sites on the E. coli Genome
Source: PLoS Genet. 2016 May 12;12(5):e1006025. doi: 10.1371/journal.pgen.1006025 (PMC4865107; doi:10.1371/journal.pgen.1006025)
Supplement: S3 Fig — A) Analysis of the Topo IV nonspecific binding. Normalized enrichment (Average number of reads in a 1kb sliding window divided by the total amount of reads) of each flag immuno-precipitation experiment was plotted as a function of the genomic position. Left panel a 100 kb region near oriC (positions 4.26 to 4.36 Mb) is represented. Right panel a 100 kb region around dif (positions 1.55 to 1.65 Mb) is represented. B) Scatter plot of the average GC content according to parC-flag IP/Input. 60 kb sliding windows were used for GC content and IP/Input. C) Average IP/Input values were normalized for GC content. D) Null model I, a Topo IV comet follows replication forks. Illustration of the Topo IV binding kinetics under null model I described in S1 Text. The x axis in the plots represents the chromosome coordinate s, going between 0 (ori) and L (ter). The y axis represents cell cycle time. The shaded areas are the positions of the Topo IV comets (also sketched as red lines on a circular representation of the chromosome), and the numbers represent the number of bound regions per replichore. Left panel: case of non-overlapping rounds. Right panel: case of overlapping rounds, in the case where the B period starts after the termination of replication within the same cell cycle. E) Topo IV binding bias, shown by the specific Input/IP values (each normalized by total reads). This bias is not compatible with a model where Topo IV binding follows replication and persists for a characteristic period of time (purple trace). (PDF) [file pgen.1006025.s003.pdf]

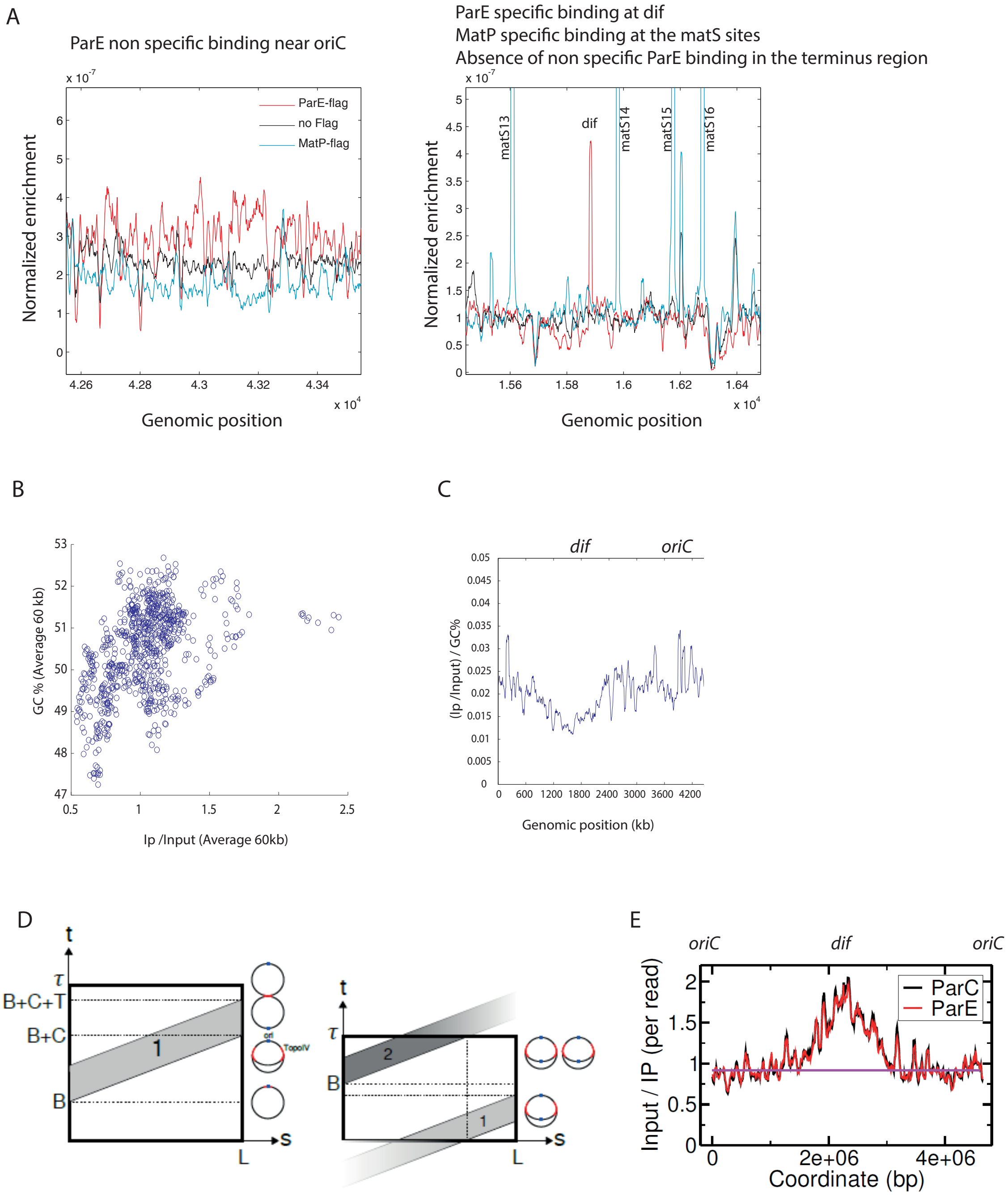

Supplementary Figure S3
