# Supplementary material for: Mapping Topoisomerase IV Binding and Activity Sites on the E. coli Genome
Source: PLoS Genet. 2016 May 12;12(5):e1006025. doi: 10.1371/journal.pgen.1006025 (PMC4865107; doi:10.1371/journal.pgen.1006025)
Supplement: S1 Text — To test if ParC and ParE ChIP-seq biases were related to chromosome replication we constructed in silico models The result of this null model is that in all cases (overlapping or non-overlapping rounds) the observed mean occupancy should follow the dosage. Hence the occupancy gap observed in S3E Fig in the Ter region (when occupancy is normalized by dosage) has to be interpreted as a sign that this model does not apply, at least in this region. (DOCX) [file pgen.1006025.s013.docx]

**Supplementary text S1**

To test if ParC and ParE ChIP-seq biases were related to chromosome replication we constructed *in silico* models. To set the notation, we assume that the genome length is *2L* and the replication time is C. This model assumes that a region of Topo IV binding nucleates at the origin when replication initiates and subsequently, in the fork region. Bound proteins have a persistence time *T*, and therefore the resulting process is a comet of bound Topo IV following the replication forks, which has a length *l= vRT*, where *vR = 2L/C* is the replication speed. We assume that this region grows linearly in time until it reaches this length at the origin and decreases at the terminus (see Fig. S3D).

We are interested in the mean behavior of cells in an exponentially growing population with a division rate *α = log 2/τ*, where t is the mean interdivision time. We therefore suppose that the cell age distribution (see Grant et al BMC Sys Biol 2011) is

**

In the following, we compute the Topo IV occupation dynamics over a cell cycle, which is described by a function *f(s; t)* describing the amounts of Topo IV bound in a locus with coordinate s found at time *t* *(t*$\in$*2 [0;L])* during the cell cycle. Since the process is replichore symmetric, we only need to describe one replichore, hence *s* $\in$ *[0; L],* where 0 is the origin and L the terminus of replication. Averaging the function *f(s; t)* over the age distribution *p(t)* gives the expected occupancy profile *o(s)* from an experiment sampling the whole population, to be compared with the experimentally observed occupancy.

a. Non-overlapping replication rounds.

In case of non-overlapping rounds, the replication cycle can simply be divided into three subsequent time periods: B (pre-initiation), C (replication), and D (post-replication). The Topo IV comet is found only during the C-period, and follows the dynamics illustrated in Figure S3. We assume that *T* is small compared to C, the duration of the replication period. In this case, Figure S3D readily shows that the occupancy function is just

where *χ(a; b)* is the characteristic function of the interval *[a; b].* Averaging over cell age gives

It is simple to verify that this function for the average expected occupancy is simply proportional to population-mean dosage, which is known to be

Indeed, in both functions the only term dependent on s is proportional to *2^-(Cs/τL)^* multiplicative constants.

b. Overlapping replication rounds. Such a case is illustrated in the right panel of Figure S3D.As the sketch illustrates, the function *f(s; t)* is slightly more complex in this case. The difference is that, because of the overlapping rounds, multiple Topo IV comets can form on different genome copies. However, averaging over cell age *p(t)* gives the exact same function *o(s)* as for the case of non-overlapping rounds above. The reason is that lower frequency of older cells always compensates for the effect of multiple copies in the same way for total dosage and for comet number.

Conclusions

The result of this null model is that in all cases (overlapping or non-overlapping rounds) the observed mean occupancy should follow the dosage. Hence the occupancy gap observed in Figure S3E in the Ter region (when occupancy is normalized by dosage) has to be interpreted as a sign that this model does not apply, at least in this region.
